# Diverse feather shape evolution enabled by coupling anisotropic signalling modules with self-organizing branching programme

**DOI:** 10.1038/ncomms14139

**Published:** 2017-01-20

**Authors:** Ang Li, Seth Figueroa, Ting-Xin Jiang, Ping Wu, Randall Widelitz, Qing Nie, Cheng-Ming Chuong

**Affiliations:** 1 Department of Pathology, University of Southern California, Los Angeles, California 90033, USA; 2 Department of Biomedical Engineering, University of California, Irvine, California 92627, USA; 3 Department of Mathematics, University of California, Irvine, California 92697, USA; 4 Integrative Stem Cell Center, China Medical University, Taichung 404, Taiwan; 5 Center for the Integrative and Evolutionary Galliformes Genomics, National Chung Hsing University, Taichung 402, Taiwan; 6 Research Center for Developmental Biology and Regenerative Medicine, National Taiwan University, Taipei 10617, Taiwan

## Abstract

Adaptation of feathered dinosaurs and Mesozoic birds to new ecological niches was potentiated by rapid diversification of feather vane shapes. The molecular mechanism driving this spectacular process remains unclear. Here, through morphology analysis, transcriptome profiling, functional perturbations and mathematical simulations, we find that mesenchyme-derived *GDF10* and *GREM1* are major controllers for the topologies of rachidial and barb generative zones (setting vane boundaries), respectively, by tuning the periodic-branching programme of epithelial progenitors. Their interactions with the anterior–posterior WNT gradient establish the bilateral-symmetric vane configuration. Additionally, combinatory effects of *CYP26B1*, *CRABP1* and *RALDH3* establish dynamic retinoic acid (RA) landscapes in feather mesenchyme, which modulate *GREM1* expression and epithelial cell shapes. Incremental changes of RA gradient slopes establish a continuum of asymmetric flight feathers along the wing, while switch-like modulation of RA signalling confers distinct vane shapes between feather tracts. Therefore, the co-option of anisotropic signalling modules introduced new dimensions of feather shape diversification.

## Abstract (web summary)

Asymmetric feather vane shape was a critical innovation in feather evolution and adaptation for flight. Here, Li and colleagues characterize the multi-module regulatory network that controls feather vane shape and underlies feather diversification.

Over the last two decades, spectacular palaeontological discoveries, mainly from China, have revolutionized our understanding in the origin and evolution of feathers^1^,^2^,^3^,^4^,^5^,^6^,^7^,^8^. Major novel functions of feathers that evolved include endothermy, communication, aerodynamic flight and so on. These are achieved through stepwise retrofitting of the original feather forms^1^,^2^,^3^,^7^,^8^.

The three major transformative events that occurred during feather shape evolution are: (i) singular cylindrical filaments to periodically branched feathers; (ii) radially symmetric feathers to bilaterally symmetric feathers by developing mirror-imaged vanes separated by a central shaft (rachis) and (iii) symmetric or asymmetric alterations of vane shapes, including the innovation of feathers specialized for flight. Previous comparative analysis of flight feather (remige) shapes in a variety of birds indicates a strong association between the level of vane asymmetry and flying ability^9^. These feathers serve as mini-airfoils that can generate lift. The co-localization of the centre of gravity and the centre of the lifting force in these feathers make the birds more stable in the air. These feathers also facilitate unidirectional pass-through of air during flapping. Additionally, they can separate from each other to minimize wind resistance^9^,^10^,^11^,^12^,^13^,^14^,^15^. Besides these major transformative events, other morphologic features that emerged during evolution include the deep follicles containing stem cells for cyclic regeneration^7^, the hooklets and curved flanges in barbules and the solid cortex and air-filled pith in rachis and ramus^16^. Together, these features enhanced feather mechanical strength, reduced weight, improved air-trapping efficiency and ensured renewability of feathers after damage.

In the past, efforts have been made to unveil the patterning rules and molecular circuitries generating different feather forms. For the previously mentioned transformative event (i), BMP and its antagonist, NOGGIN, were shown to regulate branching periodicity^17^. An activator/inhibitor periodic-branching (PB) model was further used to explain how branching morphogenesis occurs autonomously by interactions of diffusible morphogens in the epithelium^18^. For event (ii), feather stem cells were found to exhibit a ring configuration, horizontally placed in downy feathers but tilted downward anteriorly (rachis side) in bilaterally symmetric feathers^19^. An anterior–posterior *WNT3A* gradient was shown to convert radial to bilateral feather symmetry. Flattening of the gradient converted bilaterally to radially symmetric feathers^20^. Yet for event (iii), it remains unclear how feather vane shapes are altered in different body regions (for example, symmetric body plumes vs asymmetric remiges along the wing), at different growth phases (for example, primary remiges of large flying birds have naturally occurring emarginated notches, meaning different vane widths at different phases of feather growth). Understanding of feather polymorphism at different physiological developmental stages (for example, natal down and adult plumes) and across different genders (for example, sail-shaped remiges occur in male but not female mandarin ducks) is also lacking. We believe studying the complex feather vane shapes in Aves provides great opportunities to understand how systematic and environmental information are sensed and interpreted by skin appendage stem cells.

Here through anatomic and computational analysis we found two morphological parameters highly associated with feather vane shape diversity: the topology of the barb generative zone (BGZ) and the insertion angles of barbs into the rachis. The BGZ is where the regularly spaced barbs initiate and hence it has also been called the new barb locus^21^. Morphologically it is thinner than the neighbouring epithelial regions, containing irregularly spaced small branches. Eventually it disintegrates to allow vanes to separate and the feather cylinder to open up upon feather maturation.

Through transcriptome profiling and functional perturbations, we identify mesenchyme (pulp) derived *GDF10* and *GREM1* as key regulators for rachis and BGZ topology, respectively. They function by modulating BMP signalling in adjacent epithelium. The interaction between WNT signalling, *GDF10* and *GREM1* establishes the symmetric vane configuration. Additionally, differentially localized *CYP26B1*, *CRABP1* and *RALDH3* in the pulp establish anisotropic RA signalling. This modulates *GREM1* expression and epithelial cell shapes which then adjusts BGZ topology and the barb-rachis angle, resulting in alterations of vane width and symmetry. Thus the co-option of multi-scale mesenchymal signalling modules by feather epithelial progenitor cells likely drives vane shape diversification during feather evolution.

## Results

### Morphological traits affecting feather vane width/asymmetry

**Figure 1:**
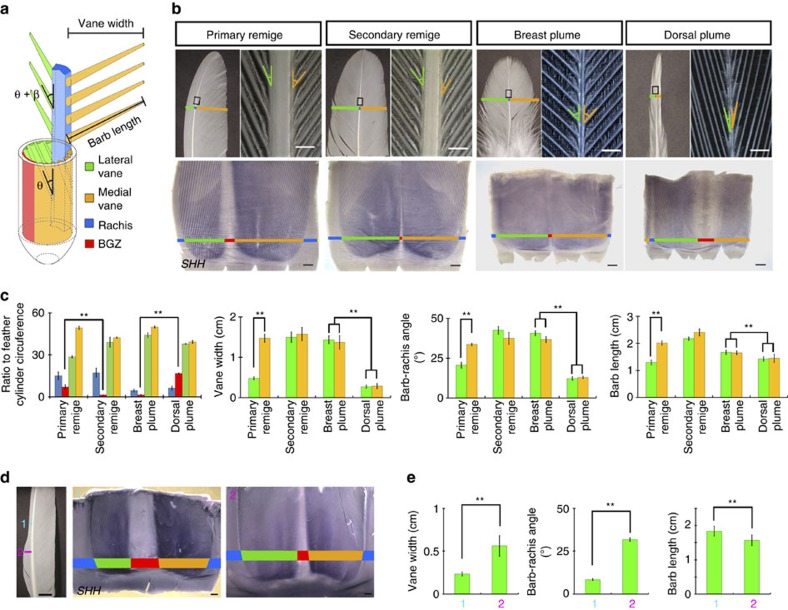
Feather vane shapes vary with differential BGZ topology and barb-rachis angles. (**a**) Schematic drawing of feather before and after maturation. The barb-rachis angle is a combination of the helical growth angle (θ) during branching morphogenesis and the expansion angle (β) after maturation. (**b**) Comparing morphologies of two types of chicken remiges with different asymmetry levels and two types of body plumes with different vane widths before and after maturation. Feather epithelial cylinder (before maturation) was cut open at the rachis side and *SHH in situ* hybridization was used to highlight vanes. Boxes indicate the regions magnified on the right. Vane widths and barb-rachis angles are highlighted by bars and angle symbols, respectively. Scale bars, 500 μm. (**c**) Quantification of the ratio of different feather epithelial regions before maturation (*n*=4); vane widths, barb-rachis angles and barb lengths after maturation (*n*=30). Error bars denote s.d. **P<0.01. (**d**) Emarginated primary remiges demonstrate abrupt change of vane width. Feathers growing to a point ∼1 cm distal to the emarginated notch position were collected and compared with those ∼1 cm proximal to the notch. A significant shrinkage of the BGZ was observed in those proximal to the notch. Scale bars, 500 μm. (**e**) Comparisons of vane widths (*n*=10), barb-rachis angles (*n*=16) and barb lengths (*n*=16) at positions ∼1 cm distal and proximal to the notch in mature feathers. Error bars denote s.d. **P<0.01.

We started by analysing the morphology of rooster remiges with different asymmetry levels (primary and secondary remiges) and body plumes with different vane widths (dorsal and breast plumes) (Fig. 1). Before feather maturation, the pulp (considered to be the source of nutrition during growth) is enwrapped by epithelium. This, in turn, is wrapped inside the feather sheath which gives the feather primordium a cylinder conformation. Upon maturation the pulp retreats and the BGZ disintegrates, allowing the vanes to separate (Fig. 1a and Supplementary Movie 1). Before feather maturation, by cutting open the feather epithelial cylinder at the rachis side and removing the pulp, we find that the width of the BGZ is inversely correlated with the width of the vanes (*SHH* has enriched expression between barbs^22^ and hence was used as a vane marker). For asymmetric remiges, higher asymmetry levels are associated with a larger BGZ that expands toward the lateral side, restricting the epithelial area for lateral vane formation (Fig. 1b,c). For symmetric body plumes, narrower vanes are associated with a larger BGZ which expands in both lateral and medial directions. More interestingly, when we examine BGZ topology in remiges with emarginated vanes, we also find abrupt vane width changes associate with significant BGZ size alterations (Fig. 1d). In contrast, the feather cylinder diameter barely changes during the emergence of the emarginated notch (Supplementary Fig. 1a,b).

Another morphological parameter associated with vane shape variation is the barb-rachis angle. It is a combination of helical growth angle during feather branching morphogenesis and the expansion angle after feather maturation^23^ (Fig. 1a). The expansion angle is likely determined by the physical properties of barbs and barbules^21^. Our measurement shows narrower vanes have smaller helical growth angles and smaller barb-rachis angles (Fig. 1b,c, Supplementary Fig. 1c–f). As to barb length, notable differences are observed between the asymmetric vanes of primary remiges (Fig. 1c). However, the differences between dorsal and breast plumes are very minor (Fig. 1c), and the wider vane region in emarginated remiges even have slightly shorter barbs (Fig. 1e). Thus vane width and barb length are not always positively correlated with each other.

### Identifying crucial molecules for vane shape regulation

**Figure 2:**
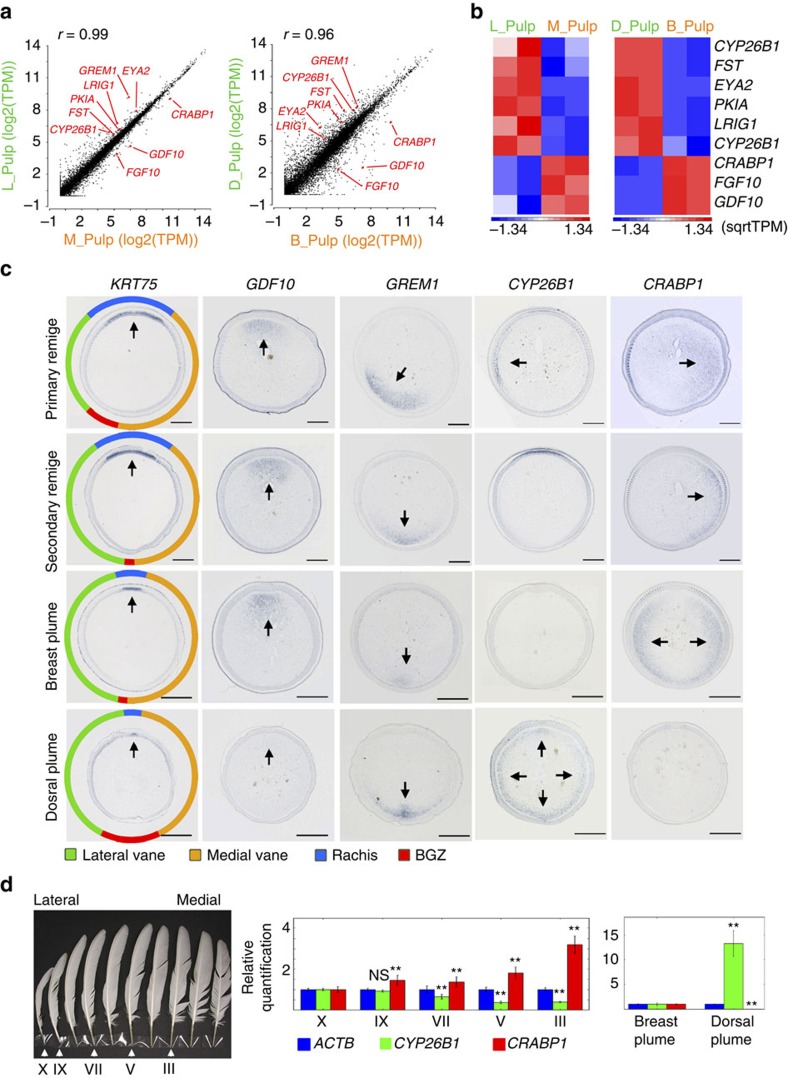
Identifying key molecular regulators of feather vane shapes. (**a**) Scatter plots depicting the transcriptomic comparisons between lateral and medial primary remige pulp, dorsal and breast plume pulp, respectively. Among the differentially expressed genes we picked out crucial signalling molecules (red) for further characterization. The linear correlation coefficient (*r*) is very close to 1, indicating highly similar transcriptome profile between samples. (**b**) The highlighted genes are clustered in two groups, one associated with narrower vanes and the other associated with wider vanes (L and M are the lateral side and medial side of primary remige; D, dorsal plume; B, breast plume, respectively. Two biological replicates are shown). (**c**) Candidate genes highlighted by RNA-seq analysis demonstrated differential localization in the pulp of growing feathers with different vane shapes (arrows). *KRT75* is highly expressed in the rachis epithelium and hence was used as a marker for position alignment between samples. Scale bars, 500 μm. (**d**) qPCR for *CYP26B1* and *CRABP1* in the pulp of primary remiges along the wing (lateral-to-medial: X, IX, VII, V, III, *n*=3 for each position) demonstrates gradually decreased *CYP26B1* expression and increased *CRABP1* expression. While dorsal plumes have more strikingly elevated *CYP26B1* and downregulated *CRABP1* expression compared with the breast plumes (*n*=3). Error bars denote s.d. **P<0.01, NS, not significant.

To screen for crucial molecules that regulate vane shapes, we performed RNA-seq to compare transcriptomes between the ‘narrow-vane' group (lateral side of primary remiges, dorsal plumes) and the ‘wide-vane' group (medial side of primary remiges, breast plumes) from roosters (Fig. 2a,b, Supplementary Fig. 2). Feather epithelium and pulp were separated during preparation for tissue-to-tissue comparisons. We also performed Histone 3 Lysine 4 tri-methylation ChIP-seq to confirm the differential expression revealed by RNA-seq, as H3K4me3 is an epigenetic marker of actively transcribed genes^24^ (Supplementary Fig. 3). Because transcriptome profiles showed high similarity between samples of the same tissue type (for example, linear correlation coefficient *r*=0.99 for lateral vs medial pulp samples, Fig. 2a) we applied the following conditions for candidate screening rather than the statistical test provided by analysis of variance: minimum fold change >1.8; minimum transcripts per kilobase million of the group with higher gene expression >25; the direction of change is identical for both members of the same group (Supplementary Tables 1–4).

From this screening, we chose crucial morphogenetic signalling molecules for further characterization by *in situ* hybridization, so we can observe the spatial distribution of these molecules. For example, RNA-seq analysis indicated that *GDF10*, a BMP family member, is downregulated in the pulp of lateral side primary remiges (2.15-fold) and dorsal plumes (37.1-fold) (Fig. 2a,b, Supplementary Table 1). *In situ* hybridization unveiled highly localized *GDF10* expression in the pulp adjacent to the rachis (Fig. 2c). RNA-seq also indicated that the BMP antagonists, *GREM1* and *FST,* are upregulated in the pulp of lateral side primary remiges (7.30-fold, 1.93-fold) and dorsal plumes (2.12-fold, 4.68-fold) (Fig. 2a,b, Supplementary Table 2). *In situ* hybridization demonstrated localized *GREM1* expression in the pulp adjacent to the BGZ (Fig. 2c). *FST* does not display such a localized pattern (Supplementary Fig. 4). Similar expression patterns of *GDF10* and *GREM1* were also seen in zebra finch and Japanese quail remiges (Supplementary Fig. 5).

Furthermore, two genes along the RA pathway: *CYP26B1*, which degrades RA (ref. 25), is upregulated in the pulp of lateral side primary remiges (2.98-fold) and dorsal plumes (2.64-fold), while *CRABP1*, which shuttles RA either to the nuclear receptors (RARs) or degrading enzymes^26^, is downregulated in the pulp of lateral side primary remiges (10.22-fold) and dorsal plumes (1.93-fold) (Fig. 2a,b, Supplementary Tables 1 and 2). *In situ* hybridization also demonstrated complementary expression patterns of *CYP26B1* and *CRABP1* (Fig. 2c). *CRABP1* is colocalized with the RA synthetase, *RALDH3,* in breast plumes but no differential expression of RA receptors was detected (Supplementary Fig. 4). Immunostaining revealed nuclear-enrichment of CRABP1 protein in pulp cells (Supplementary Fig. 6). Nuclear CRABP1 implies its potential role in trans-activating nuclear RA receptors^27^. Therefore, CRABP1 protein likely promotes RA signalling in the pulp and this also implicates the presence of a lateral–medial RA signalling gradient in primary remiges.

Interestingly, quantitative PCR (qPCR) for *CYP26B1* and *CRABP1* in the pulp of primary remiges along the wing (lateral-to-medial: X, XI, VII, V, III) demonstrated gradually decreasing *CYP26B1* expression (0.96-fold, 1.68-fold, 2.41-fold, 2.59-fold compared with that of X) and increasing *CRABP1* expression (1.45-fold, 1.38-fold, 1.81-fold, 3.2-fold compared with that of X), implying position-dependent, gradual alteration of RA gradient slope in primary remiges. Meanwhile dorsal plumes have more prominently elevated *CYP26B1* (13.34-fold) and downregulated *CRABP1* expression (215.77-fold) compared with the breast plumes (Fig. 2d), implicating switch-like regulation of RA signalling regulation between these two feather tracts.

### *GDF10* is a crucial modulator of rachis topology

**Figure 3:**
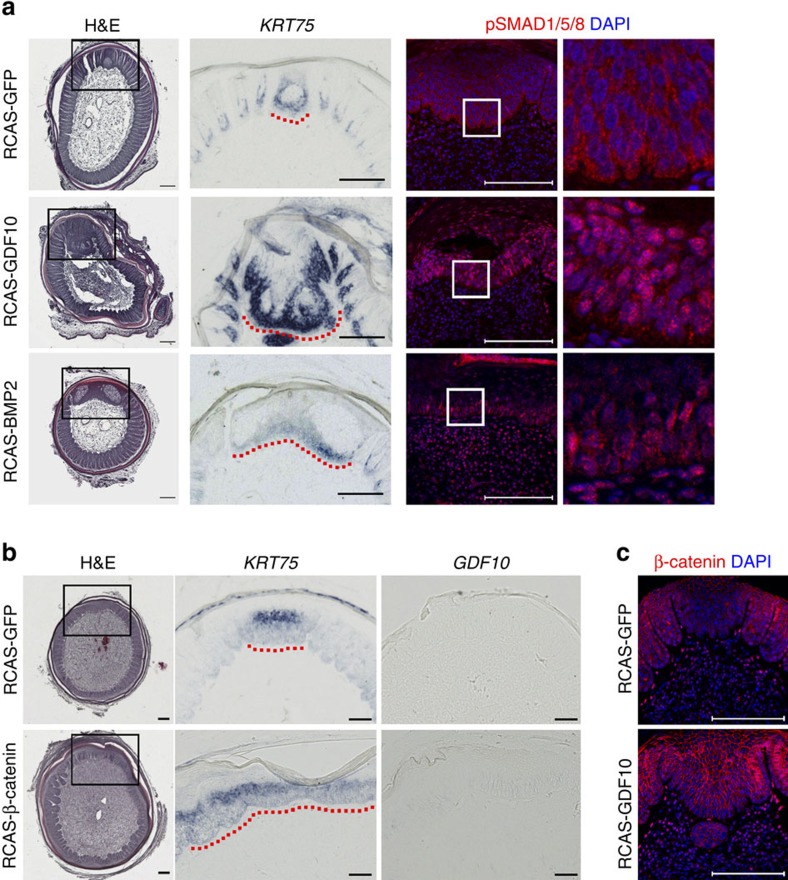
*GDF10* is a crucial modulator of rachis topology. (**a**) Compared with the RCAS-GFP (control) infected neonatal primary remiges, *GDF10* and *BMP2* mis-expressing feathers developed enlarged rachises as shown by Hematoxylin & Eosin (H&E) staining and *KRT75 in situ* hybridization. Nuclear pSMAD1/5/8 positive cells also increased in the rachis region. Dotted red lines highlight the rachis. Scale bar, 100 μm. (**b**) RCAS-β-Catenin infected neonatal remiges developed expanded rachis without notable upregulation of *GDF10* expression. Dotted red lines highlight the rachis. (**c**) RCAS-GDF10 infection increased nuclear β-Catenin positive cells in the pulp adjacent to the rachis. Scale bar, 100 μm.

Due to the high correlation between the *GDF10* expression pattern and the rachis topology in different feather types, we investigated whether there is a cause and effect relationship between the two. Our attempts to clone full length chicken *GDF10* were unsuccessful. This made us speculate that the transcripts might be spliced. Hence we did a 5′-RACE experiment (Rapid amplification of 5′ cDNA ends) and found the majority of *GDF10* transcripts are spliced at Exon 2, which matched the spliced ESTs reported on the UCSC Genome Browser (Supplementary Fig. 7). We cloned the 3′ part of the spliced transcript into the replication competent avian sarcoma (RCAS) viral vector and infected embryonic chicken limbs. Three out of five of the neonatal primary remiges developed more prominent rachises (Fig. 3a). We previously found that *BMP2* mis-expression caused an enhancement of rachis formation^17^. Therefore, we examined BMP signalling activity in the *GDF10* mis-expressing follicles. Indeed, we observed an increased number of nuclear phosphorylated (p)-Smad1/5/8 positive cells, indicating active BMP signalling (Fig. 3a).

Previous studies also indicated that WNT signalling plays a crucial role for the formation of bilaterally symmetric feather vanes^20^, so we also examined the potential crosstalk between *GDF10* and WNT signalling. Neonatal primary remiges infected with virus mis-expressing constitutively active β-Catenin developed expanded rachises without significant upregulation of *GDF10* expression (Fig. 3b). It is worth noting that unlike adult chicken primary remiges, neonatal primary remiges have very thin rachises and barely detectable endogenous *GDF10* expression. On the other hand, in normal feathers we observe abundant nuclear β-Catenin positive cells in the pulp adjacent to the rachis and vanes, but not in the BGZ region. This regional difference potentially results from the anterior–posterior epithelial WNT gradient, because in body plumes with after-feathers (secondary rachis and vanes forming at the original BGZ region) upregulated *WNT3A* expression and increased numbers of nuclear β-Catenin positive pulp cells are observed in the original BGZ region (Supplementary Fig. 8a). Interestingly, in neonatal primary remiges infected with RCAS-GDF10, the number of nuclear β-Catenin positive pulp cells also seems to increase (Fig. 3c). Therefore, beside BMP signalling, *GDF10* may potentially modulate the spatial activity of WNT signalling. But WNT signalling itself can function without upregulation of *GDF10*.

### *GREM1* specifies BGZ topology

Due to the high correlation between the *GREM1* expression pattern and the BGZ topology in different feather types we investigated whether there is a cause and effect relationship between the two. The antagonistic property of *GREM1* on BMP signalling also makes it a key candidate molecule for BGZ specification because a previous mathematical model depicting feather shaping highlighted a crucial role for BMP inhibition in the initiation of barb formation^18^.

**Figure 4:**
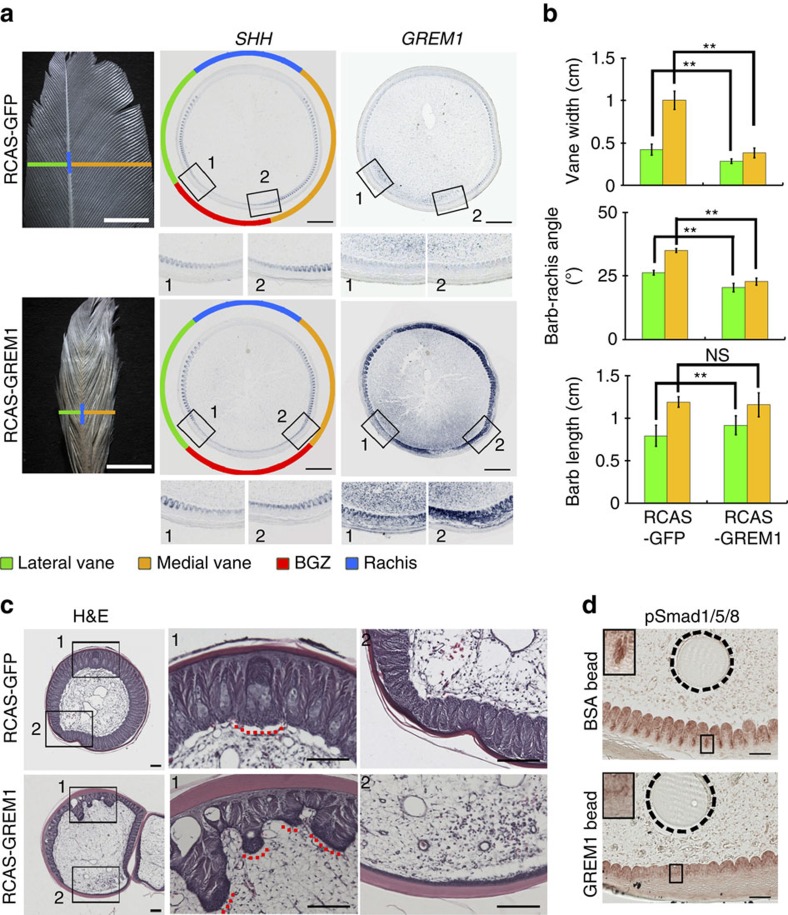
*GREM1* is a key regulator of BGZ topology. (**a**) Compared with the controls, *GREM1* mis-expressed adult chicken primary remiges have reduced vane width and increased BGZ width. Boxes indicate the vane boundaries between BGZ and vanes magnified below. Scale bars: 5 mm for the leftmost panel, 500 μm for the right two panels. (**b**) Comparing vane widths (*n*=4), barb-rachis angles and barb lengths (*n*=10) in control and *GREM1* mis-expressed feathers. Error bars denote s.d. **P<0.01, *P<0.05, NS, not significant. (**c**) RCAS-GREM1 infected neonatal remiges not only had an expanded BGZ but also a branched rachis. Boxed regions are magnified on the right. Dotted red lines highlight the rachis. Scale bar, 100 μm. (**d**) GREM1 soaked beads reduced nuclear pSMAD1/5/8 staining in the neighbouring epithelial cells. Boxed regions are magnified on the top-left corner. Dashed lines highlight the beads. Scale bar, 100 μm.

To evaluate the functional role of *GREM1*, we injected RCAS viral vectors carrying *GREM1* (ref. 28) or control (GFP) into follicles of plucked primary remiges. Ten out of fifteen regenerated feathers showed altered degrees of asymmetry and mild decreases in the barb-rachis angle, while barb length barely changed (Fig. 4a,b). Sectioning the proximal follicle of feathers with phenotypic changes before maturation revealed expansion of the BGZ range compared with controls (Fig. 4a, Supplementary Fig. 9a). Furthermore, two out of three neonatal chicken remiges infected with RCAS-GREM1 developed not only a notably expanded BGZ, but also branched rachises (Fig. 4c). Hence *GREM1* promotes the feather epithelial progenitors to adopt the BGZ fate and antagonizes the rachis fate.

To confirm *GREM1* functions through antagonizing epithelial BMP signalling, we inserted GREM1 protein coated beads (1 mg ml^−1^) into the pulp next to the vane region in growing body plumes. Three out of four samples demonstrated a BGZ-like phenotype (Supplementary Fig. 9d) and decreased nuclear pSMAD1/5/8 positive cells in the adjacent epithelium (Fig. 4d). We also examined the distribution of nuclear p-Smad1/5/8 positive cells in normal body plumes. These cells are present in the rachis and vane epithelium, but not in the BGZ epithelium, which is adjacent to the *GREM1* positive zone (Supplementary Fig. 10a). Thus *GREM1* does antagonize BMP signalling in feather epithelium.

Because the BGZ region is usually thinner than other epithelial regions during feather growth, we speculate that *GREM1*'s impact on feather epithelium includes changing the cell proliferation rate. Through PCNA staining, we indeed observe decreased epithelial cell proliferation in *GREM1* mis-expressed feather follicles compared with controls (Supplementary Fig. 9b,c). Consistent with this, we observe fewer proliferating cells in BGZ epithelium compared with other epithelial regions in most of the feather forms we examined (Supplementary Fig. 10b). Breast plumes are an exception, potentially because their endogenous *GREM1* expression is very low, or because the BGZ is so narrow that parts of the vane region were included in the region of interest used to count the number of proliferating cells.

We also investigated the potential crosstalk between *GREM1* and WNT signalling. Inserting WNT3A protein (100 μg ml^−1^) soaked beads into the BGZ pulp of growing dorsal plumes downregulated *GREM1* expression (*n*=3, Supplementary Fig. 11a). Consistently, RCAS-β-Catenin infected neonatal remiges also demonstrate decreasing *GREM1* expression (Supplementary Fig. 11b). Thus WNT signalling plays an inhibitory role on *GREM1* expression.

### Anisotropic RA signalling modulates vane width and symmetry

**Figure 5:**
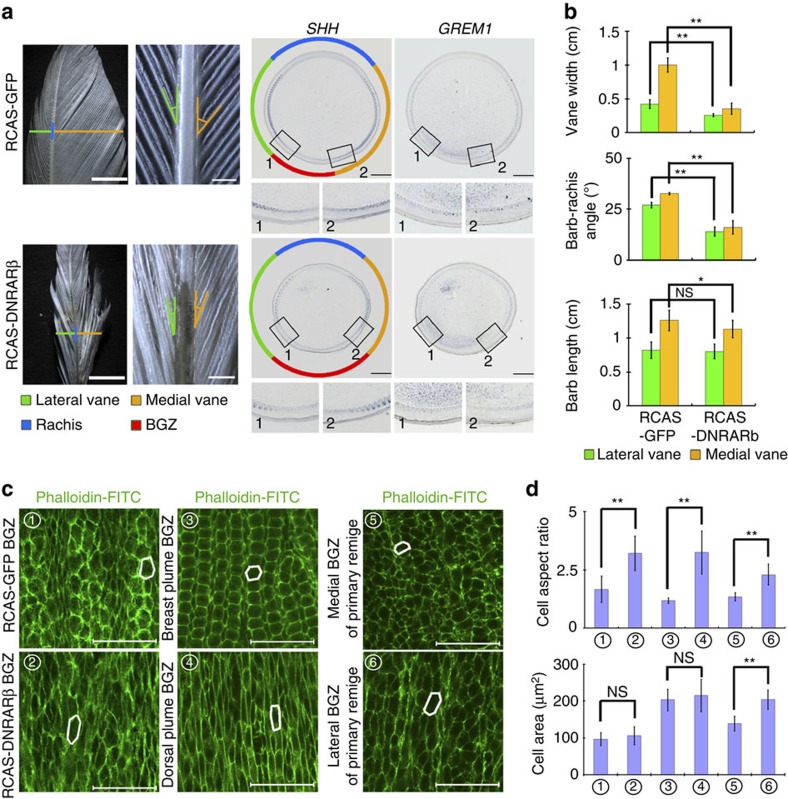
RA signalling modulates vane morphology and epithelial cell shapes. (**a**) Compared with the controls, RCAS-DNRARβ infected adult chicken primary remiges have significantly reduced vane width and barb-rachis angles, while BGZ width is increased. Boxes highlight vane boundaries. Scale bars: leftmost panel: 5 mm, right three panels: 500 μm. (**b**) Quantification of feather vane widths (*n*=4), barb-rachis angles and barb lengths (*n*=10) in control and DNRARβ infected remiges. Error bars denote s.d. **P<0.01, *P<0.05, NS, not significant. (**c**) BGZ epithelial cells have more elongated shape at epithelial regions exposed to lower RA levels. Phalloidin labels F-Actin that is mainly localized along epithelial cell boundaries. Scale bar, 50 μm. White lines highlight cell shape differences. (**d**) Quantification of cell aspect ratio and cell area in BGZ epithelial cells (*n*=30). Error bars denote s.d. **P<0.01, *P<0.05, NS, not significant.

The differential localization of RA related factors in asymmetric remiges implies a potential link between the RA gradient and vane asymmetry. When we mis-express a dominant negative form of chicken RARβ (to block wild-type RAR function^29^) in primary remiges we observe decreases in feather vane widths and significantly sharper barb-rachis angles. Meanwhile the barb length barely changes (Fig. 5a,b) (12/12). Interestingly, epithelial cells (especially those in BGZ regions) in growing remiges mis-expressing DNRARβ have a more elongated appearance (in the proximal–distal direction) than the controls (Fig. 5c,d). A similar situation is also seen in feather epithelial cells exposed to different endogenous RA levels. For example, dorsal plumes and lateral side primary remiges have upregulated *CYP26B1* in the pulp adjacent to epithelium. Their BGZ epithelial cells are more elongated than those in breast plumes and the medial side of primary remiges, which have upregulated *CRABP1* (Fig. 5c,d). Through mathematical modelling, we identify two potential mechanisms through which cell shapes can affect the helical growth angle, which is a part of the barb-rachis angle. First, we found helical growth angle is an arctangent function of the feather elongation rate divided by the travelling speed of the activator for feather branching (Supplementary Fig. 12a). Since cell shape elongation increases the feather elongation rate, it would reduce the helical growth angle (Supplementary Fig. 12b). Second, elongated cell shape increases tissue tortuosity (Supplementary Fig. 12c,d), which can decrease the activator/inhibitor diffusivity if the diffusion occurs mainly through the epithelial cellular plane. The slower travelling wave-speed of the branching activator produces a sharper helical growth angle (Supplementary Fig. 12d). But altering the travelling wave-speed of the inhibitor barely has any effects (Supplementary Fig. 12e). These modelling results indicate that activator diffusivity and feather elongation rate, both of which are influenced by cell shape, can control helical growth angles.

Previously the *in situ* hybridization of RA related factors in different types of feathers implies an association between increased RA levels and decreased *GREM1* expression (Fig. 2c). Consistent with this, in body plumes with after-feathers, the decrease of *GREM1* at the original BGZ coincides with increased *RALDH3* and *CRABP1* (promoting RA signalling, Supplementary Fig. 8b). Meanwhile in primary remiges with emarginated notches, the reduction of *GREM1* expression (after the abrupt expansion of vane width) coincides with a decrease of *CYP26B1* (degrading RA) expression on the lateral side (Supplementary Fig. 13). Furthermore, the remiges infected with RCAS-DNRARβ have relatively wider *GREM1* positive zone while those infected with RCAS-GREM1 have not changed *CYP26B1* expression patterns (Fig. 5a, Supplementary Fig. 9a). Therefore, we hypothesize RA signalling works upstream to inhibit *GREM1* expression.

**Figure 6:**
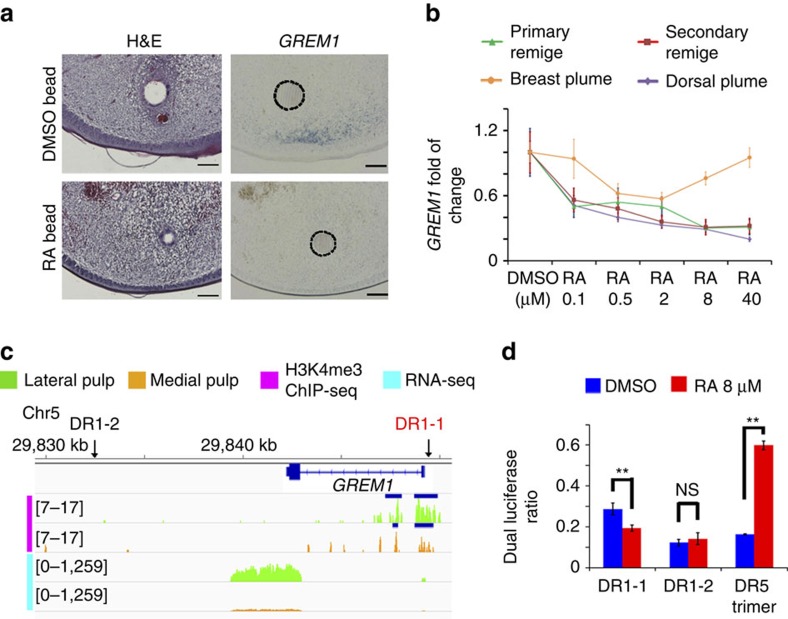
RA signalling can directly inhibit *GREM1* expression. (**a**) Insertion of AG1x8 beads soaked in 1 mg ml^−1^ RA into dorsal plumes' BGZ pulp significantly decreased *GREM1* expression compared with the DMSO treated controls. Dashed lines highlight the bead. Scale bar, 100 μm. (**b**) qPCR for *GREM1* in pulp cells from four types of feathers treated with different doses of RA (*n*=3). Error bars denote s.d. (**c**) Two conserved (chicken, turkey, zebra finch) DR1 type RAREs were identified close to the *GREM1* genomic locus. DR1-1 (highlighted in red) is in the active promoter region (H3K4me3 peak). The peak regions called by MACS are highlighted by blue bars. (**d**) DR1-1 downregulated its downstream gene expression upon RA treatment while a DR5 trimer had the opposite effect in dual luciferase assays (*n*=4). Error bars denote s.d. **P<0.01, NS, not significant.

To evaluate this possibility, we insert RA soaked beads (1 mg ml^−1^) into the BGZ pulp of growing dorsal plumes that indeed decreases *GREM1* expression compared with the control (*n*=4, Fig. 6a). Next we isolated BGZ pulp cells from different types of feathers and treated them with different concentrations of RA or a RAR antagonist. qPCR revealed a RA dose-dependent decrease in *GREM1* expression and upregulation of *GREM1* upon RAR antagonist treatment (Fig. 6b, Supplementary Fig. 14). However, cells from breast plumes behave differently, potentially due to their low endogenous *GREM1* expression (Fig. 2c). A similar trend of expression changes was also observed for *GDF10* in cultured rachis pulp cells, but the fold change is less significant (Supplementary Fig. 14).

To further examine if RA acts on *GREM1* directly, we searched for RA response elements (RAREs) close to the *GREM1* genomic locus conserved in chicken, turkey and zebra finch (Supplementary Tables 5 and 6). Using a dual luciferase assay, we show that a direct repeat 1 (DR1) type of RARE in the *GREM1* promoter region repressed reporter transcription upon RA treatment (Fig. 6c,d), consistent with previous reports of DR1 RARE's inhibitory role on gene expression upon RA binding^30^. Meanwhile the positive control with trimeric DR5 RARE demonstrated significantly increased promoter activity upon RA treatment. Hence RA signalling can directly modulate *GREM1* expression.

### A multi-module regulatory model for feather diversification

**Figure 7:**
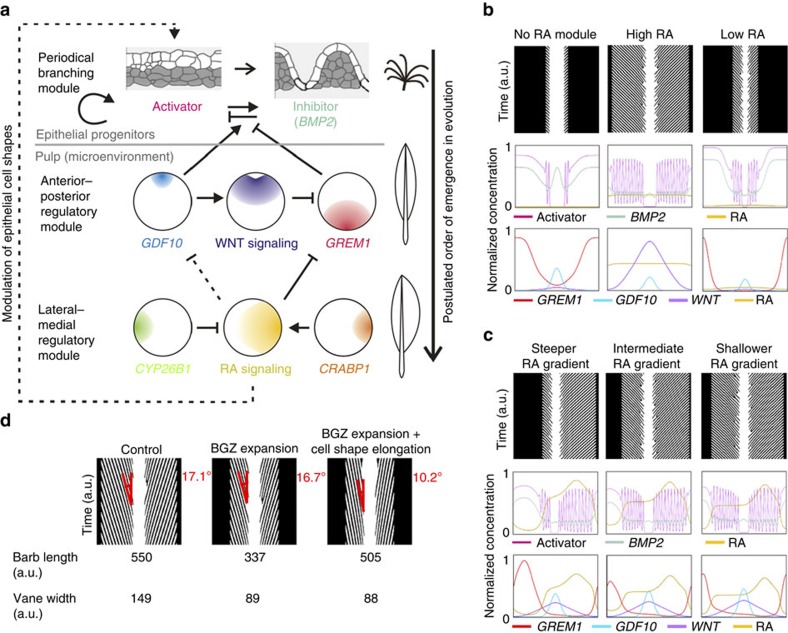
A multi-module regulatory model of feather diversification. (**a**) Schematic representation of the infrastructure of the multi-module regulatory feather (MRF) model and the corresponding transformative events of feather shapes in evolution. Dashed lines denote crosstalk relationships not fully confirmed. The differential equations for quantifying the crosstalk relationships are listed in the Methods. (**b**) Representative simulations of vane shape variations using the MRF model either without RA module, with high RA (*CRABP1* level set at 1.1, *CYP26B1* at 0.02, artificial unit), or with low RA (*CRABP1* at 0.005, *CYP26B1* at 0.2). (**c**) Representative simulations of feathers with different levels of vane asymmetry by changing the slope of RA gradient (*CRABP1* at 0.006, *CYP26B1* at 5 for the steeper RA gradient, *CRABP1* at 0.03, *CYP26B1* at 0.5 for the intermediate RA gradient, *CRABP1* at 0.06, *CYP26B1* at 0.3 for the shallower RA gradient), (**d**) Simulations of RCAS-GREM1 and RCAS-DNRARb infected feathers in which the BGZ expands without significantly shortening of barbs.

The strength of the PB model proposed by Prum is in its adoption of the activator–inhibitor concept^18^. Here we integrate the new findings on *GDF10*, *GREM1*, RA and WNT signalling and established a multi-module regulatory feather (MRF) model (Fig. 7a) that provides a molecular basis for the diverse feather forms.

In this model, RA signalling is the lateral–medial regulatory module, which is mainly controlled by *CYP26B1* and *CRABP1*. The lateral–medial module then acts on the anterior–posterior regulatory module, which consists of *GDF10*, *GREM1* and WNT signalling. These two layers of regulatory modules then act together on BMP signalling, an inhibitor of barb branching^17^,^18^, leading to changes of the activator's spatial–temporal dynamics and feather branching patterns.

Mathematically, the MRF model consists of the original PB model by Prum^18^, plus 4 new reaction–diffusion equations for extracellular RA, WNT, GDF10 and GREM1. To model extracellular RA we incorporated a previous model^26^ which includes Crabp1 and Cyp26 (see ‘Methods': Model construction and simulations). We first simulate the MRF model with and without the lateral–medial regulatory module. The simulation indicates that the anterior–posterior module alone is able to produce rachis and BGZ at opposite sides of the feather cylinder. When a symmetric lateral–medial module is introduced, the feather vanes can enlarge or shrink based on the level of RA, which is modulated by *CYP26B1* and *CRABP1* (Fig. 7b). The MRF model can also simulate bilateral asymmetric feathers by establishing opposing gradients of *CYP26B1* and *CRABP1* in the lateral–medial direction. Through incrementally changing their expression in the opposite direction, which changes the slope of the RA gradient, a continuum of asymmetry levels could be produced (Fig. 7c). Such a continuum of asymmetry is commonly observed in a row of remiges along the alar tract on the bird wing (Fig. 2d, Supplementary Fig. 5)

## Discussion

Organ shaping is a fundamental issue in development and critical in tissue engineering. In many cases organ shapes are influenced by signals arising both within and outside the organ. We believe the diverse feather vane shapes in modern birds provide a great opportunity to decipher the principles of morphogenesis and understand how stem cells can alter their behaviours in response to different environmental information.

Here we established a multi-module regulatory model revealing that the feather mesenchyme provides micro-environmental signals to tune the self-organized branching programme of feather epithelial progenitors. First, branching of the feather epithelial cylinder requires interactions between activators and inhibitors. *BMP* signalling appears to be the major inhibitor^17^,^18^*. SHH*, *NOGGIN* and *SPRY4* have been considered as the candidate activators^17^,^18^,^31^. However, there has not been any evidence showing enforced expression of *SHH* can induce ectopic feather branching. *NOGGIN* and *SPRY4* could enhance feather branching but their spatial distribution does not fit the prediction from the PB model. Therefore, the molecular identity of the activator may remain to be revealed. Second, *GDF10* and *GREM1* acted on BMP signalling to tune the branching process, leading to the establishment of Rachis and BGZ topology, respectively. Third, a WNT gradient coordinated the position of the rachis and BGZ through interactions with *GDF10* and *GREM1*, which established the bilateral-symmetric vane configuration. Fourth, the anisotropic RA landscape, shaped by differential levels of *CYP26B1*, *RALDH3* and *CRABP1* over different body regions and time, introduced a new dimension of vane shape variations through crosstalk with *GREM1* to adjust the BGZ topology (and potentially *GDF10* to adjust rachis topology) and barb-rachis angles.

Differential RA signalling activities have been implicated as a key regulator of region-specific phenotypes. For example, *RALDH2*^*−/−*^ mouse embryos have been reported to show bilaterally asymmetric somitogenesis due to left–right desynchronization of segmentation clock oscillations^32^. Previous analyses of naked neck chickens revealed elevated RA in the neck potentiates BMP signalling, which inhibits feather formation^33^. Our findings here indicate *GREM1* as a potential candidate to explain the potentiation effect of RA on BMP signalling. During limb development, RA forms a gradient for proximal–distal limb patterning^25^, which is in the same direction as those in primary remiges. Therefore, it is possible that the limb RA gradient is somehow imprinted within the remige pulp cells and the steepness of the limb RA gradient is used to establish a continuum of asymmetry levels in remiges along the wing.

Beside the gradual changes of RA gradient, we also observed switch-like regulation of RA signalling in breast vs dorsal plumes. This may result in the differential expression of Homeobox genes or T-box genes, which are body axis organizers that can potentially regulate *GREM1* expression^34^,^35^,^36^. Additionally, RA signalling can be swiftly modulated during feather growth to give rise to abrupt vane shape changes like emarginated notches.

Besides its effect on BGZ topology, RA signalling also modulates barb-rachis angles, which may further contribute to the diversification of vane shapes. A recent publication indicates the emergence of asymmetric remige vanes and the differential barb-rachis angles between vanes occurred at different times during evolution, and it is the differential barb length that determines vane asymmetry^37^. In Mesozoic birds such as *Archaeopteryx and Confuciusornis,* the narrower lateral vanes of primary remiges have shorter barbs but comparable barb-rachis angle to the wider medial vanes. In our work, we found the feather vane width and barb length are not always correlated in chicken feathers (Fig. 1c,e). Therefore, it is possible that the differential barb-rachis angles were evolved after the Mesozoic period.

Then how could RA signalling modulate barb-rachis angles at the molecular level? We observe different feather epithelial cell shapes in feather vanes having different RA levels. Lower RA is associated with a more elongated cell shape in the proximal–distal direction (Fig. 5c). Through mathematical modelling we demonstrate two potential mechanisms through which cell shapes could affect helical growth angles (Supplementary Fig. 12), and the helical growth angle is part of the barb-rachis angle. Interestingly, in RCAS-GREM1 infected feathers we observed narrower vanes with negligible changes of barb length (Fig. 4b), which could be explained by a compensation effect from sharper helical growth angles (Fig. 7d). Since RA inhibits *GREM1* expression, the sharper helical growth angle and elongated cell shapes in RCAS-DNRARb infected feathers may also due to elevated *GREM1* level. Consistent with this, when we compare chicken dorsal and breast plumes, the dorsal plumes have higher *GREM1* expression accompanied by sharper barb-rachis angles and helical growth angles (Fig. 1c, Supplementary Fig. 1e,f), as well as more elongated epithelial cell shapes (Fig. 5c). It is also worth noticing that the barb length is only slightly different between dorsal and breast plumes (Fig. 1c). These results are also consistent with a previous report that the barb length is affected by both the relative position of the BGZ to the rachis and the helical growth angles^23^. In other systems, RA signalling also has been shown to influence cell shape/polarity^38^,^39^. Cell shape polarization is usually related to planar cell polarity (PCP) signalling^40^,^41^. Meanwhile the non-canonical WNT/PCP pathway has also been implicated in establishing body bilateral asymmetry by regulating the distribution and movement of cilia^42^. Key PCP pathway members were not highlighted by our RNA-seq analysis. However, PCP proteins endow cell polarity through their asymmetric localization within cells, which would not be detected by transcriptome analysis.

Besides the barb-rachis angle and the topology of the BGZ, there are other parameters that can modulate feather vane width such as the epithelial cylinder circumference during feather growth. We believe that the gradual change of the circumference does contribute to the production of the smoothly arced contour of feathers. However, the keratinized follicle and feather sheath and the tension from extra-follicular musculature may limit its contribution to abrupt vane width changes such as the formation of emarginated notch.

In summary, our study here reveals a multi-module regulatory network in feather mesenchyme that facilitates the diversification of feather vane shapes through regulating the branching morphogenesis of feather epithelial progenitors. Such interactions between organ stem cells and their microenvironment are also observed in the development and regeneration of other organs^43^. It would be intriguing to investigate how these microenvironment signals crosstalk with systematic signals, such as differential hormone levels in different genders, seasons and physiological developmental stages.

## Methods

### Animals

Animal care and experiments were conducted according to the guidelines established by the USC Institutional Animal Care and Use Committee. Pathogen free Charles River (Connecticut, USA) white leghorn chickens were used for virus injection and feather follicle collection.

### Measurements of feather anatomic parameters

For normal remiges and body plumes, the measurements of barb-rachis angle, barb length and vane width were done in the vane area 3 cm and 2 cm below the distal tip, respectively. For RCAS virus injected adult chicken remiges, the measurements were done 1–2 cm below the tip. To collect growing feathers proximal and distal to the emargination notch, we carefully measured the distance of the notch to the feather tip for primary remige VI–VIII for each chicken and collect the regenerating feather at least 1 cm shorter than that distance. The sample proximal to the notch is the regenerating feather ≥1 cm longer than the distance.

### RNA-seq and ChIP-seq sample preparation and analysis

For RNA-seq we cut the proximal follicles of three primary remiges from Spafas white leghorn chickens into the lateral and medial halves and separated the epithelium from the mesenchyme in 2xCMF solution. For dorsal plumes and breast plumes, 25 proximal follicles were used, respectively. Total RNA was extracted using Trizol reagent. A total of 4 μg RNA was used for library construction by TruSeq RNA Sample Prep Kit Version2 (Illumina). Two biological replicates were sent for sequencing (50 bp, single end) with Illumina HiSeq 2000 in the USC Epigenome Center. FastQ files were trimmed and mapped to the chicken genome (galGal4) using Partek Flow. Further analysis was done in Partek Genomic Suite. For ChIP-seq, 15 primary remige follicles were collected and processed similar to those used for RNA-seq. The mesenchymal cells were dissociated by 0.35% Collagenase digestion before formaldehyde cross-linking. The ChIP experiments were performed as described previously^44^, including the formaldehyde cross-linking of cells for 14 min at room temperature followed by sonication for 20 min (30 s on/off cycle). Immunoprecipitation with H3K4me3 antibodies were performed at 4 °C overnight. After cross-link reversal, DNA from two biological replicates were sent for library preparation and sequencing by the USC Epigenome Center. Reads were trimmed and aligned to galGal4 in Partek Flow. Peak calling was done with MACS. Results were viewed in the Integrative Genomics Viewer.

### Whole-Mount and section RNA *in situ* hybridization

Whole-Mount *in situ* hybridization was performed as in Jiang *et al*.^45^. Briefly, samples were fixed in 4% paraformaldehyde for 2 days, followed by PBS washing, dehydration, and rehydration. Hybridization with digoxigenin labelled probes took place in a 65 °C water bath overnight. The samples were washed and incubated with anti-digoxigenen-AP antibodies at 4 °C overnight. Colour development was implemented with Promega NBT/BCIP substrate. Proximal feather follicles were cut open at the rachis side and the mesenchyme was removed in 2xCMF solution to expose the inner side of the epithelial cylinder. Samples were kept flat during probe hybridization in chambers with narrow gaps. Section *in situ* hybridization was done as described^46^. Samples were fixed and washed following Whole-Mount protocols. After dehydration, the tissue was then embedded in paraffin and sectioned at 8 μm. Sections were rehydrated, acetylated before hybridization with digoxigenen labelled probes overnight at 65 °C. Antibody incubation and colour development followed Whole-Mount protocols. The *SHH* and *KRT75* probe have already been described^22^,^47^. Primers for probe cloning are listed as follows: c-*RALDH3*: gcccatcaaggtgtgttctt and tgccaaagcatattcaccaa; c-*CRABP1* sense: acctggaagatgaggagcag and ggtcacatacaacaccgcatt; c-*CYP26B1*: cctgatagagagcggcaaag and gttggaatccagtccgaaga; c-*EYA2*: cattcccggcctaactgtgt and agcatgtaactgcagggtcc; c-*FGF10*: aatggtgcctcagcctttt and ccattggaagaaagtgagctg; *c-FST*: aggaggacgtcaacgacaac and atcgacctctgccaacctta; c-*GDF10*: tcccatactgaggctcaacc and tgttttcgccttgctttctt; c-*GREM1*: cgacagcagaagggagaaag and gcacttctcggcttagtcca; c-*KRT75*: atgtctcgccagtccaccg and ttagctcctgtaacttctcc; c-*RARα*: acggagtgctcggagagtta and tgcagtttgtccaccttgtc; c-*RARβ*: acttgttccaagccctcctt and tgcatctgagttcggttcag; *c-SHH*: ggaattcccag(ca)gitg(ct)aa(ag)ga(ag)(ca)(ag)i(gct)tia and tcattiatggaccca(ga)tc(ga)aaiccigc(tc)tc; zebra finch-*GDF10*: cgagctggagagctcattct and gacaatcttgggcattggaaa; zebra finch-*GREM1*: catcggtgccttgtttcttc and gacaccggcactccttaactc; zebra finch-*SHH*: tctcctctgggctgacttgt and cgccactgagttttctgctt; *In situ* hybridization in quail samples used chicken probes.

### RT-qPCR of cultured pulp cell RNAs

The pulp from the most proximal part (5 mm) of growing feathers was separated from epithelium by brief 2xCMF treatment, then either directly processed for RNA extraction or dissociated in 0.35% Type I Collagenase for culture. The cultured (48 h) pulp cells were with different drug treatments: RA (Sigma R2625), RAR antagonist (ER 50891 Tocris 3823). RNA was isolated with the Qiagen RNeasy Mini Kit and the concentration was measured with the NanoDrop 2000 spectrophotometer. Reverse transcription was done with Superscript III (Life Technologies). qPCR primers: *ACTB*: gctatgaactccctgatggtc and ggactccatacccaagaaaga; *CRABP1*: ccacggaaatcaacttcaaaa and ttgttttcattctcccaggtg; *CYP26B1*: tgcccctgtctttaaggatg and aggggaggtagtggaacctg; *GREM1*: tcttctgacgggatttctgc and aatcattgggctgatccttg. *GDF10*: tgtgctgagcttgattctgg and ttgcaaggtcattggcataa; Maxima SYBR Green/ROX qPCR Mix was from Thermo Scientific. The Ct values were measured by Agilent Mx3000P qPCR system. The relative quantification was done by pyQPCR v0.9 software. Each condition has at least two biological replicates and three technical replicates.

### Rapid amplification of 5′ cDNA ends and cloning

Rapid amplification of 5′ cDNA ends was done using the GeneRacer core kit and Superscript III RT module following the manual (Invitrogen). Overall, 2 μg primary remige pulp RNA was used as the starting material. The gene specific primer for *GDF10* is cggcaggcacaagtttcaactgacat. The PCR product was amplified using the GeneRacer 5′ Nested Primer and the gene specific primer was ligated with pDrive vector. We transformed DH5α cells with the ligation mixture and used blue/white screening for colonies with insert for sequencing.

### Identifying candidate RARE and Dual luciferase assay

Search of RARE close to the *GREM1* genomic locus was done in the Genomatix Software Suite. The two candidate DR1 type RA-response elements were cloned into pGL3-SV40 Promoter Vector (no enhancer). pRL-SV40 Vector (with enhancer) serves as the internal control. Both vectors are from P. C. Tang at National Chung Hsing University. pGL3-RARE-luciferase (Addgene #13458) was used as the positive control. Primary remige BGZ pulp cells were co-transfected with the pGL and pRL vectors for 6 h using Lipofectamine 2000 (Life Technologies) and then switched to media containing DMSO or RA for another 1.5 days. The cells were then processed with the Promega Dual-Luciferase Reporter Assay System and the luciferase intensity was read by Perkin Elmer EnVision Multilabel Plate Reader at the USC Translational Research Laboratory.

### Immunohistochemistry and immunofluorescence

*Antibodies*. pSMAD1/5/8 (Millipore, AB3848); β-Catenin (Sigma, C-2206); Phalloidin-FITC (Sigma,P-5282); CRABP1 (Abcam, ab2816); PCNA (DAKO, clone PC10); CldU (Abcam, ab6326). Immunostaining follows our published method^45^. All samples are fixed in paraformaldehyde for 2 days at 4 °C and washed. For phalloidin staining, samples were pre-blocked and incubated with Phalloidin-FITC for 1 h at room temperature. For Whole-Mount immunostaining, samples were dehydrated, rehydrated, pre-blocked and incubated with the primary antibody at 4 °C overnight. They were then incubated with a secondary antibody conjugated to Alexa Fluor 488 (green) or Alexa Fluor 594 (red) labelled for 6 h. For section immunostaining, antigen retrieval was carried out through citrate buffer in a 95 °C water bath. Primary antibody incubation is at 4 °C overnight and secondary antibody incubation is at room temperature for 3 h.

### CldU labelling and quantification

To show transient amplifying cells, chickens were injected with the BrdU homologue, CldU (Sigma) at 5 mg kg^−1^. Feathers were collected 2 h later. To quantify the CldU positive cell ratio, six circles (100 μm diameter) were drawn on the confocal image in different regions. CldU positive and DAPI stained nuclei within the circles were quantified using ImageJ.

### Virus injection and bead implantation

RCAS-GREM1 is a gift of J.-C. Belmonte, Salk Institute. The primers for cloning GDF10 isoforms are: ggggacaagtttgtacaaaaaagcaggcttccatatcataaaagaatcaaaacgatct and ggggaccactttgtacaagaaagctgggtcttatcggcaggcacaagttt. The primers for cloning RCAS-DNRARβ are: ggggacaagtttgtacaaaaaagcaggcttcattaacatgacaaccagca and ggggaccactttgtacaagaaagctgggtctcaaggaatttccattttcaacgta. The PCR products were cloned into RCAS using BP-LR reaction (Life Technologies). RCAS virus was produced by transfecting DF-1 cells with the corresponding plasmids. Supernatant was collected, ultra-centrifuged and injected as described^17^. For bead implantation, heparin bead soaked in PBS or recombinant human Gremlin protein (R&D Systems 5190-GR) for 1 h at room temperature were inserted into the peripheral pulp of growing phase contour feathers around the ramogenic zone level. The feathers were collected 24–48 h later. To achieve localized WNT signalling activation, Affi-Gel Blue beads soaked Mouse WNT3A (100 μg ml^−1^, PeproTech 315-20) were used. For delivery RA, Bio-Rad AG1X8 beads were soaked in 1 mg ml^−1^ retinoic acid (RA) for 20 min in dark.

### Confocal microscopy and image processing

A Zeiss LS510 confocal microscope was used to image the fluorescently labelled specimens. Background correction, *z*-stack compaction, image stitching and cell counting were done in Fiji ImageJ^48^,^49^.

### Statistical analysis

Two independent sample *T*-tests were done with Matlab.

### Model construction and simulations

All the simulation codes for solving the Partial Differential Equations along with analysis of tortuosity and estimation of the barb-rachis angle are developed using Matlab.

### Periodic-branching model

The PB model is based on a previous activator–inhibitor model^18^, where a diffusible self-regulating activator *A*, activates a diffusible inhibitor *B* and a non-diffusible inhibitor *C*. Both inhibitors *B* and *C* downregulate activator *A*. The GREM1 and GDF10 steady state profiles formed in the MRF model are incorporated in the PB model through inhibiting *B* downregulation of *A*, and upregulating basal production of *B*, respectively.


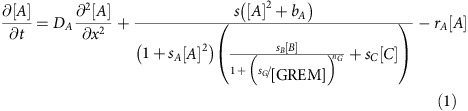



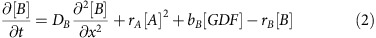



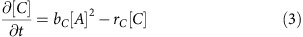


The concentration of activator *A* is represented by [*A*], inhibitors *B* and *C* by [*B*] and [*C*], Grem1 by [*GREM*] and GDF10 by [*GDF*]. Here, *D*_*A*_ and *D*_*B*_ are the diffusion coefficients of *A* and *B*, *r*_*A*_, *r*_*B*_ and *r*_*C*_ are decay rates of *A*, *B* and *C*. Parameter *s* modulates the maximum autocatalytic reaction of *A*, *s*_*A*_, *s*_*B*_, *s*_*C*_ and *s*_*G*_ are saturation coefficients, *b*_*A*_ and *b*_*B*_ are the basal production of *A* and *B*, while *b*_*C*_ modulates the maximum production of *C*. *n*_*G*_ is a Hill coefficient. Simulation parameters are listed in Supplementary Table 7.

### Prediction of helical growth angles and estimation of feather tortuosity

 


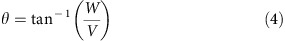


Predicted helical growth angle, *θ*, is equal to the inverse tangent of the propagating wave speed of the activator, *W*, divided by the feather elongation rate, *V*. Wave speed was estimated by calculating the product of the mean wavelength and frequency of the travelling activator waves. Wave speed can be thought of as the horizontal component of barb growth as shown in Supplementary Fig. 12a.


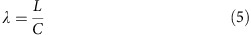


The tortuosity of the tissue, *λ*, is calculated as the length of the shortest path between two points, *L*, divided by the length of the chord connecting those two points, *C*. Supplementary Fig. 12c shows examples of calculating the path length between two points in a tissue. Mean tortuosity calculated for different feathers are shown in Supplementary Table 8.


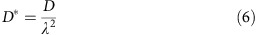


The apparent diffusivity coefficient, D*, of a molecule in a medium is equal to its free diffusivity, *D*, divided by the square of the tortuosity of the medium. Supplementary Table 8 shows the effect of tortuosity on diffusion coefficients in different feather tissues. A more tortuous medium leads to a lower apparent diffusion of a molecule.

### Multi-module regulatory feather model

In the MRF model the concentration of extracellular diffusible RA is represented by [*RA*_*o*_], diffusible WNT by [*WNT*], diffusible GDF10 by [*GDF*] and diffusible GREM1 by [*GREM*]. The concentrations of non-diffusible molecules are similarly represented, intracellular RA by [*RA*_*i*_], CRABP1 by [*BP*], CYP26B1 by [*CYP*] and RA receptors by [*R*]. [*RA*_*i*_] bound with [*R*] forms the complex [*RAR*], which we use as a RA signal, and [*RA*_i_] bound with [*BP*] forms the complex [*RABP*]. The interactions between *RA*_*o*_, *RA*_*i*_, *BP*, *CYP* and *R* are based on a previous model^26^, with the *WNT*, *GDF* and *GREM* interactions added based on our experimental result (parameters are listed in Supplementary Table 9).


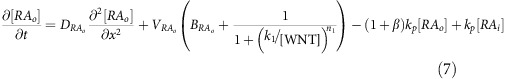



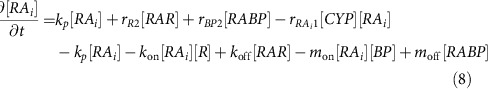



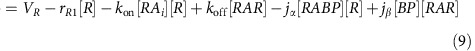



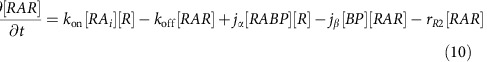



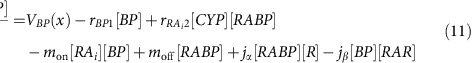



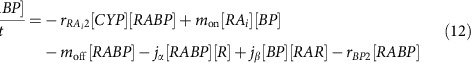



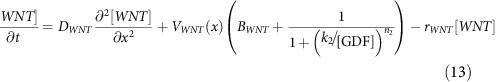



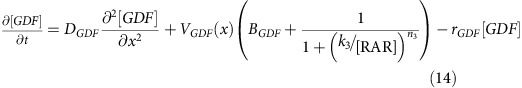



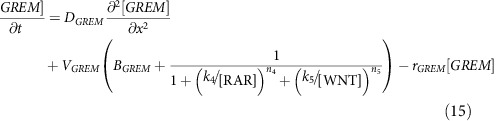


Here, *V*_*i*_, *B*_*i*_, *D*_*i*_ and *r*_*i*_ are the maximum production rates, basal production rates, diffusion coefficients and the decay rates of molecule type *i (=RA*_*o*_, *RA*_*i*,_ *R, RAR, BP, RABP, WNT*, *GDF, GREM),* respectively. In some cases, a molecule can decay when it is both unbound and bound in a complex. Therefore, two decay rates are given: *r*_*BP*1_ and *r*_*BP*2_ for CRABP1 unbound and bound to RA, *r*_*R*1_ and *r*_*R*2_ for the RA receptor unbound and bound to RA, and *r*_*RAi*1_ and *r*_*RAi*2_ for *RA*_*i*_ unbound and bound to CRABP1. *k*_on_, *k*_off_, *m*_on_ and *m*_off_, are on and off rates for complexes *RAR* and *RABP*. The rate at which RA in complex [*RAR*] unbinds and binds with CRABP1 to form [*RABP*] is *j*_*α*_, while the rate at which RA in complex [*RABP*] unbinds and binds to RA receptors to form [*RAR*] is *j*_*β*_. Diffusible extracellular RA is modelled as entering the cell at rate *k*_*p*_, and *β* is the proportion of RA lost from the system in this transition. Regulation of activation and inhibition between molecular species are regulated by Hill functions, where *k*_1_, *k*_2_, *k*_3_, *k*_4_ and *k*_5_ are dissociation constants and *n*_1_, *n*_2_, *n*_3_, *n*_4_ and *n*_5_ are Hill coefficients. Some maximum production rates, as well as the concentration of CYP26B1 are spatially dependent and are defined as follows:

Where *x*[0, 1)


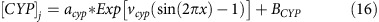



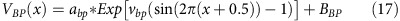



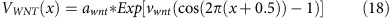



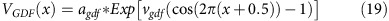


Here, *a*_*i*_ is a scaling coefficient and *v*_*i*_ defines the slope of the gradient formed.

We solved the system of partial differential equations using a second order finite difference scheme in space. For the temporal discretization, the MRF model uses a fourth order Runge–Kutta time integration method while the PB model uses a first order Euler method. The MRF model is run to steady state. Different spatial resolutions have been used to test the code for the order of accuracy of the numerical method, including *N*=200, 400 and 800, where *N* is the number of points for spatial discretization. Similarly, the temporal order of accuracy of the approximation has also been tested and studied.

### Data availability

The RNA-seq and ChIP-seq data that support the findings of this study have been deposited in NCBI GEO database with the accession number GSE86415.

## Additional information

**How to cite this article:** Li, A. *et al*. Diverse feather shape evolution enabled by coupling anisotropic signalling modules with self-organizing branching programme. *Nat. Commun.* **8,** 14139 doi: 10.1038/ncomms14139 (2017).

**Publisher's note:** Springer Nature remains neutral with regard to jurisdictional claims in published maps and institutional affiliations.

## Supplementary Material

Supplementary Information Supplementary Figures and Supplementary Tables

Supplementary Movie 1 Simulation of body plume development and maturation process. The dynamics of branching activators are shown underneath the simulation.
