# Supplementary material for: Diverse feather shape evolution enabled by coupling anisotropic signalling modules with self-organizing branching programme
Source: Nat Commun. 2017 Jan 20;8:ncomms14139. doi: 10.1038/ncomms14139 (PMC5263876; doi:10.1038/ncomms14139)
Supplement: Supplementary Information — Supplementary Figures and Supplementary Tables [file ncomms14139-s1.pdf]

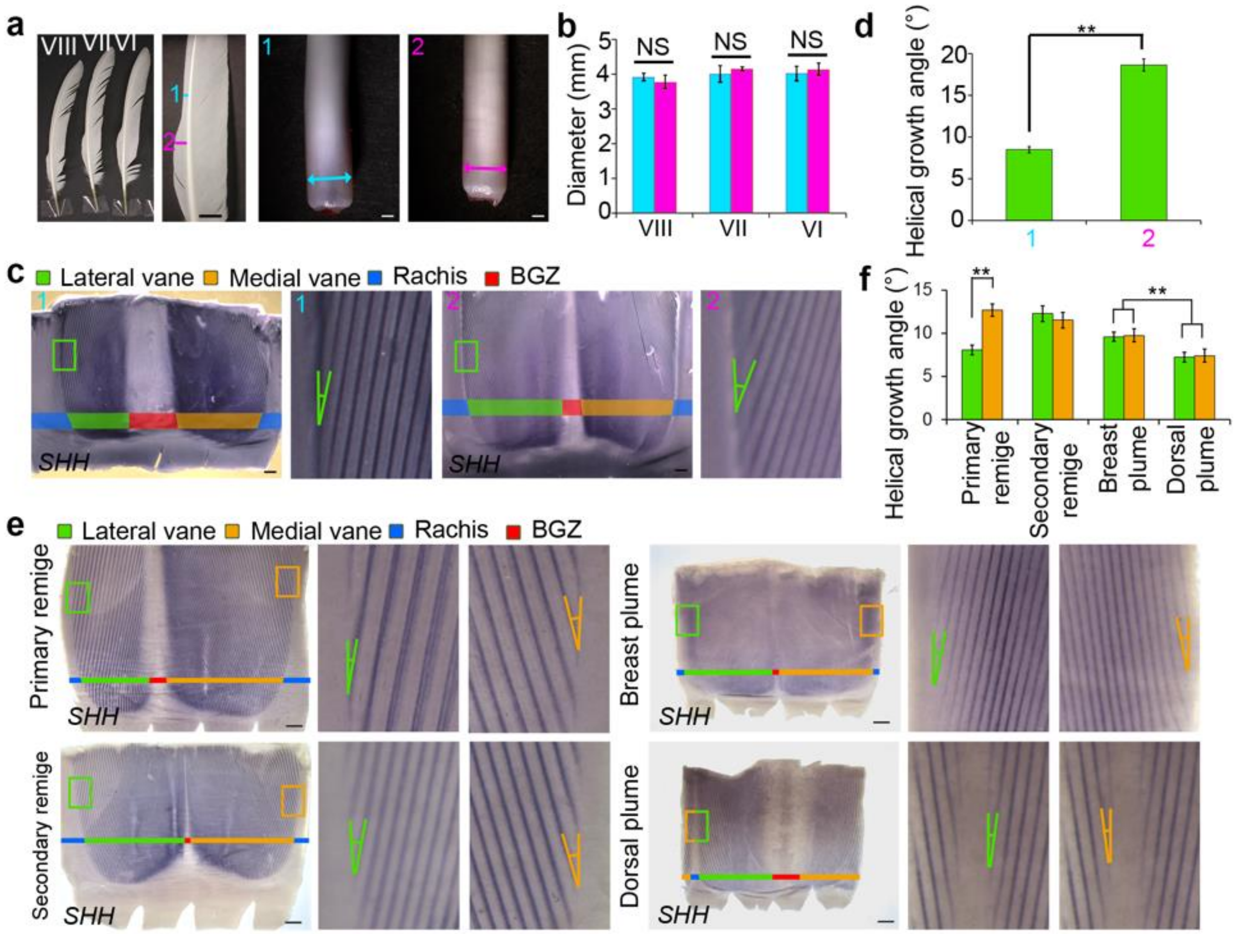

Supplementary Figure 1 | Measuring feather cylinder diameters and helical growth angles.

(**a**) Primary remiges VI-VIII have notable emargination notches and the feathers growing to a point ~1 cm distal to the emargination notch position were collected and compared to those ~1 cm proximal to the notch. Scale bars: 1cm for the left panel and 1 mm for the two right panels. (**b**) Diameters of the feather cylinders barely changed (n = 5). Error bars denote SD. NS: not significant. (**c**) The feather cylinders were cut open at the rachis side, with pulp removed and *SHH* in situ hybridization was done to highlight helical growth angles of barbs. (**d**) Significant differences of helical growth angles were observed in the vane region ~1 cm distal and proximal to the emargination notch. Error bars denote SD. ** $p < 0.01$ (n = 8). (**e**) Helical growth angles

for the lateral and medial barbs in the four different types of feathers before maturation. Scale bars: 500 µm. (**f**) Measurements of the helical growth angles ($n = 20$ for each region) demonstrated an association between sharper angles and smaller vanes. Error bars denote SD. ** $p < 0.01$.

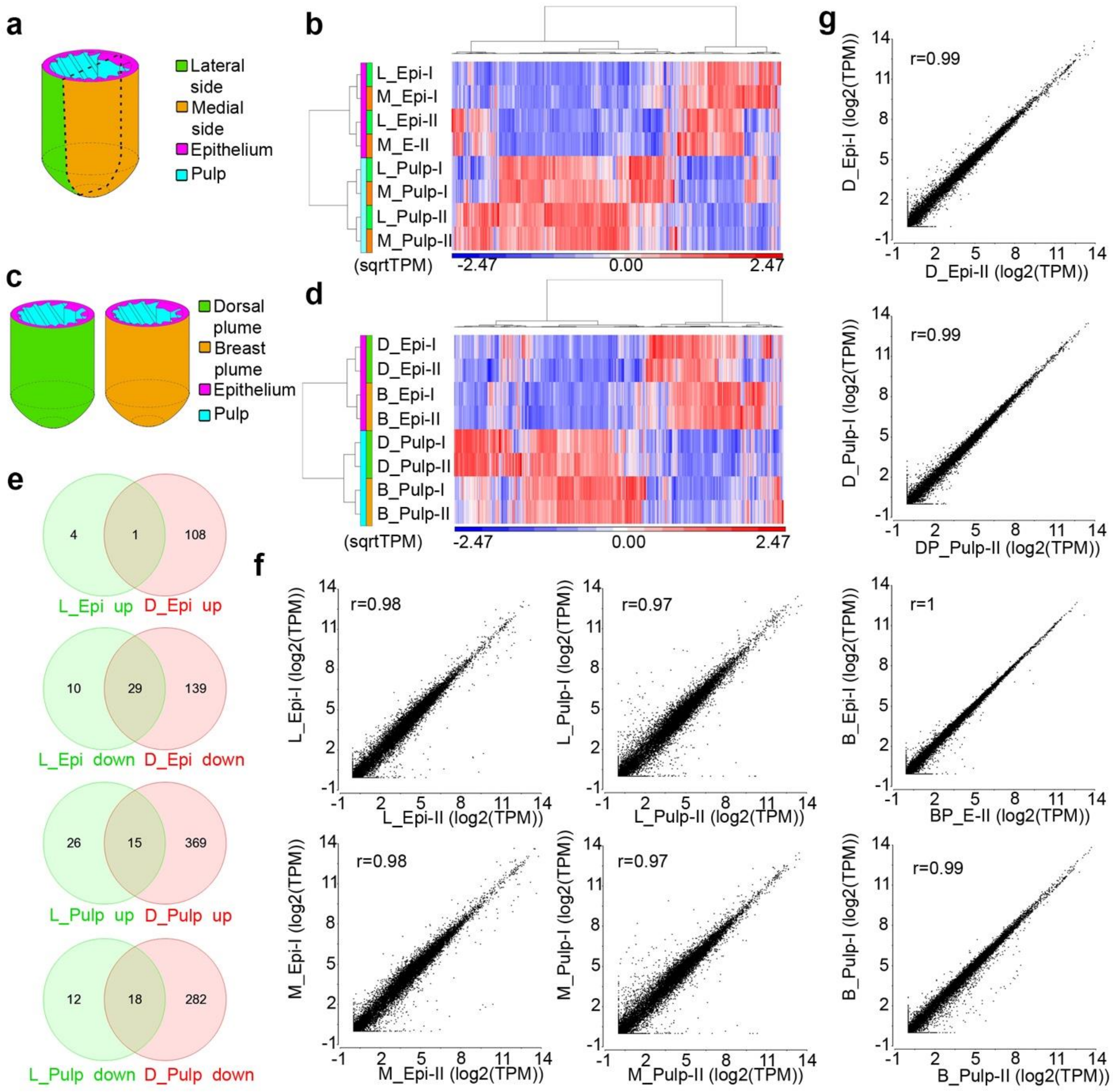

Supplementary Figure 2 | Transcriptome analyses of different feather forms. (a) Preparation of the lateral and medial side epithelium/pulp from primary remiges for RNA-seq. **(b)** Hierarchical clustering of remige samples and genes. L: lateral side, M: medial side, Epi: epithelium, I: Replicate-1, II: Replicate-2. TPM stands for Transcripts per Kilobase per Million. **(c-d)** Preparation of epithelium/pulp samples from dorsal and breast plumes for RNA-seq and the hierarchical clustering results. D: dorsal plume, B: breast plume. **(e)** Venn diagram of the

candidate genes associated with narrower vanes and wider vanes, respectively. **(f-g)** Scatter plots comparing gene expression over batches (biological replicates). The high linear correlation coefficient indicates high reproducibility between biological replicates and hence the RNA-seq results are reliable.

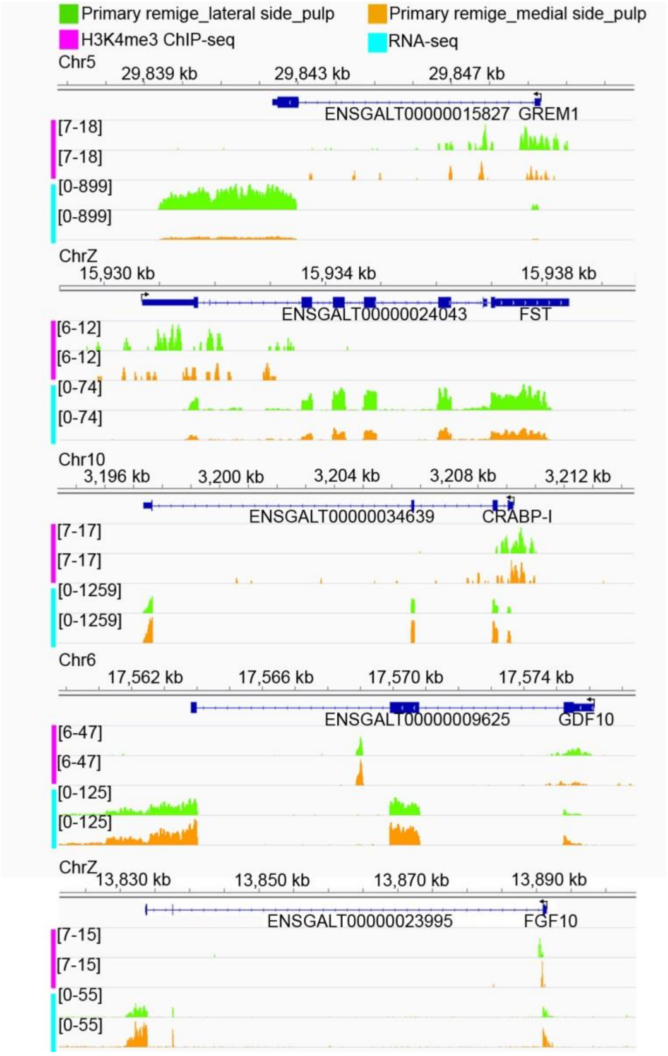

Supplementary Figure 3 | Histone3-Lysine4-trimethylation (H3K4me3) ChIP-seq analyses. H3K4me3 is a marker of actively transcribing promoters. The ChIP-seq data and RNA-seq results are generally consistent as higher gene expression associates with more prominent peaks in the promoter region. Transcription start site and orientation are indicated by arrows. Only one of the two biological replicates is shown here.

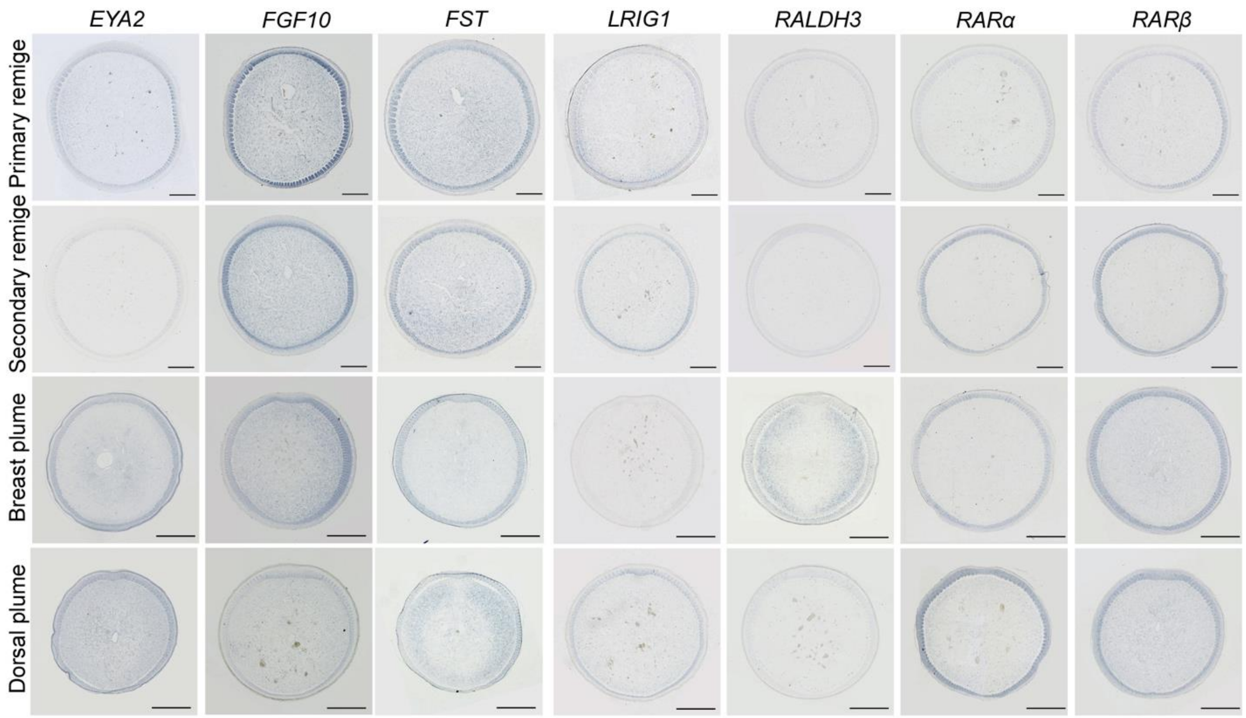

Supplementary Figure 4 | *In situ* hybridization of other candidate genes. *EYA2* and *LRIG1* have mildly up-regulated expression in the lateral pulp of primary remiges. *FST* is highly expressed in the peripheral pulp of dorsal plumes (except in the pulp region adjacent to the rachis). *RALDH3* has similar expression pattern to *FST* in breast plumes but not dorsal plume. *FGF10*, *RARα* and *RARβ* have diffuse expression patterns. Scale bar: 500 µm.

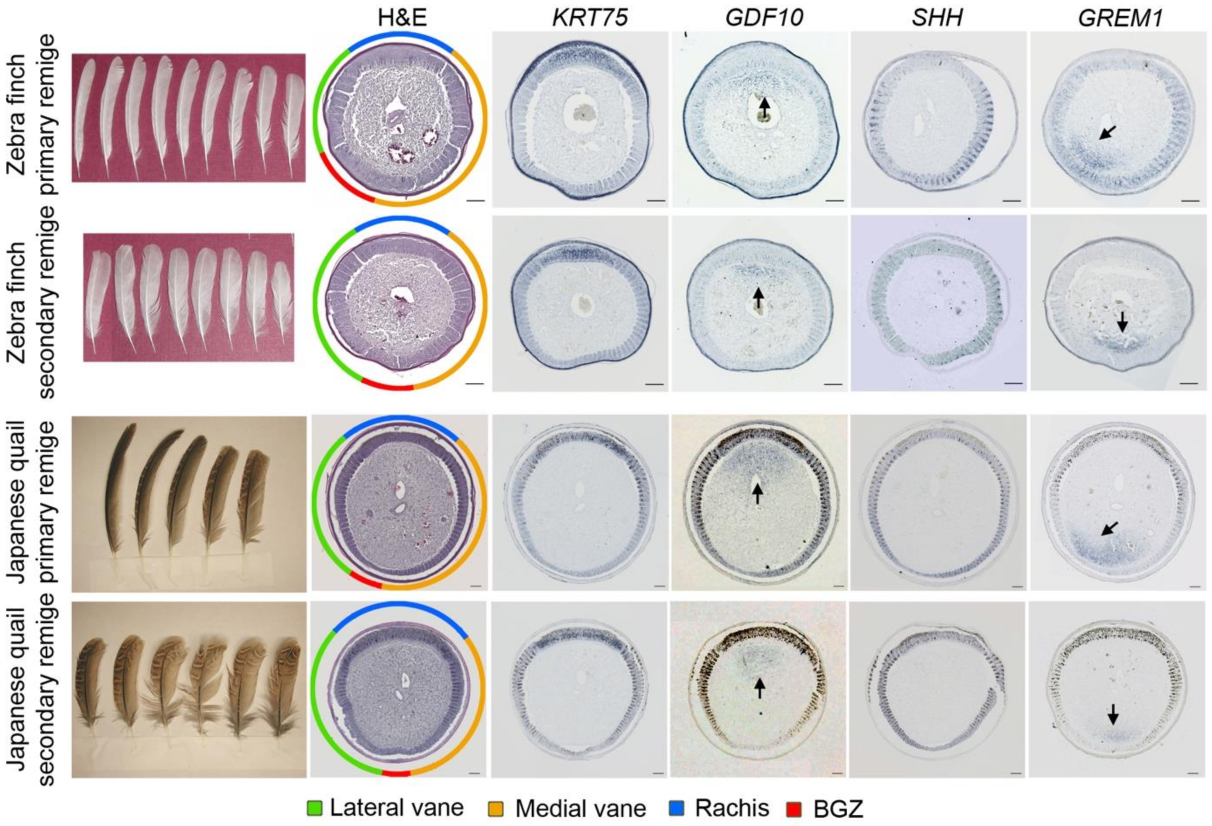

Supplementary Figure 5 | *GDF10* and *GREM1* in Japanese quail and zebra finch remiges.

Morphologies of primary and secondary remiges in Japanese quails and zebra finches, as well as the cross-sections of growing remiges with Hematoxylin & Eosin (H&E) staining, *GDF10* and *GREM1* in situ hybridization were shown. *GDF10* is localized in the pulp adjacent to the rachis and *GREM1* in the pulp adjacent to the BGZ. Scale bars: 100 µm.

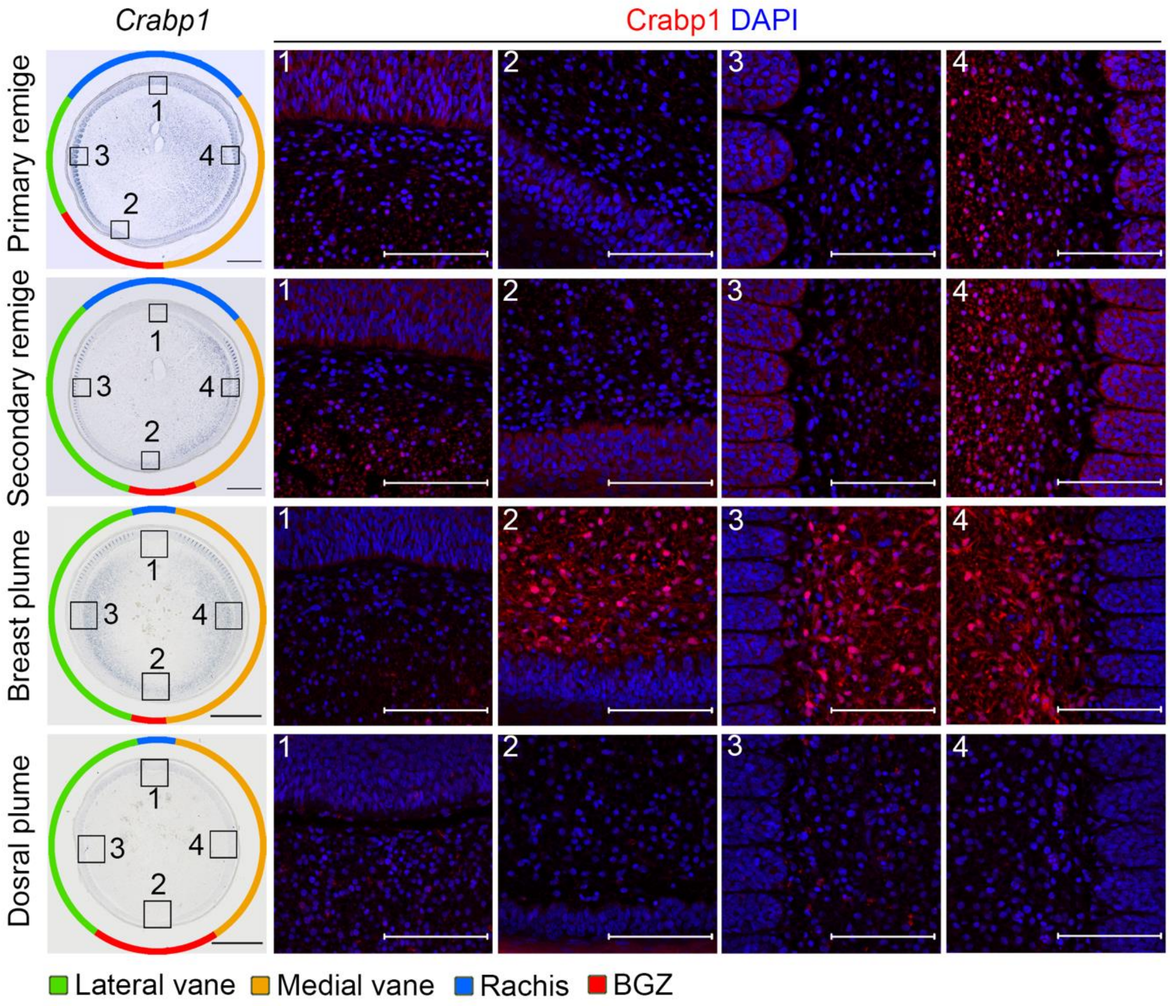

Supplementary Figure 6 | Spatial distribution of nuclear CRABP1 positive cells. Abundant nuclear CRABP1 positive cells exist in the pulp where *CRABP1* is highly expressed, such as the medial side pulp of remiges, the peripheral pulp of breast plumes (except the rachis region). Scale bar: 500 µm (leftmost panels), 100 µm (right four panels).

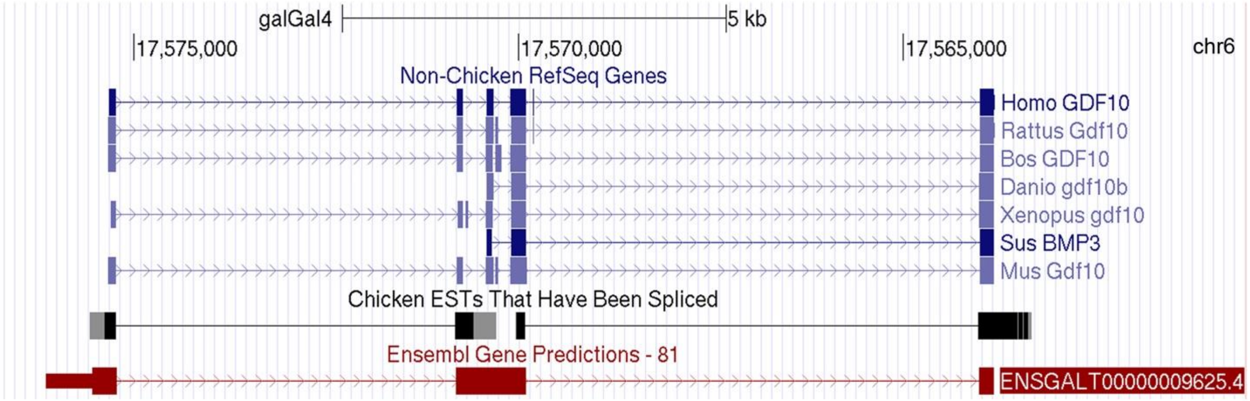

Supplementary Figure 7 | 5'-RACE of chicken GDF10 demonstrated transcript splicing.

Chicken GDF10 gene (predicted by Ensembl), the reported spliced ESTs, and homologs in other species are shown in the UCSC genome browser.

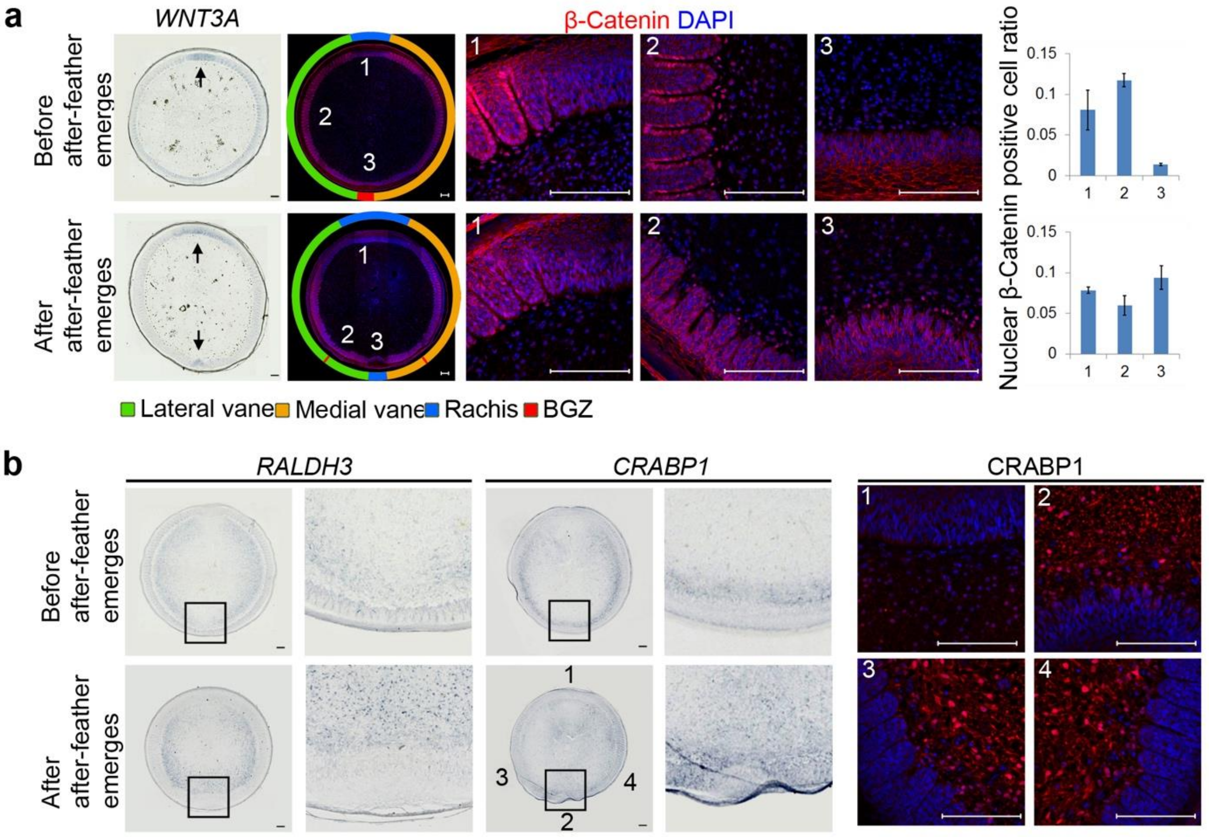

Supplementary Figure 8 | Upregulation of WNT and RA signaling in after-feathers. (**a**) In chicken breast plumes, before the after-feather emerges, *WNT3A* has enriched expression only in the rachis epithelium. Nuclear β-Catenin positive cells are present at the pulp adjacent to rachises (Zone 1), vanes (Zone 2) but not BGZ (Zone 3). After emergence of the after-feather, *WNT3A* appears in the rachis epithelium of the after-feather and the number of nuclear β-Catenin positive cells increases significantly in the neighboring pulp. The ratio of nuclear β-Catenin positive cells was calculated by dividing nuclear β-Catenin positive cell number by the number of DAPI positive cells in regions-of-interest (100 μm diameter circles, n = 6 for each zone). Scale bar: 100 μm. Error bars denote SD. (**b**) Expression of *RALDH3* and *CRABP1* increases in the pulp adjacent to the original BGZ region after the emergence of the after-feather. Nuclear CRABP1

positive cells are abundant in the pulp adjacent to after-feather rachis and vanes. Scale bar: 100 µm.

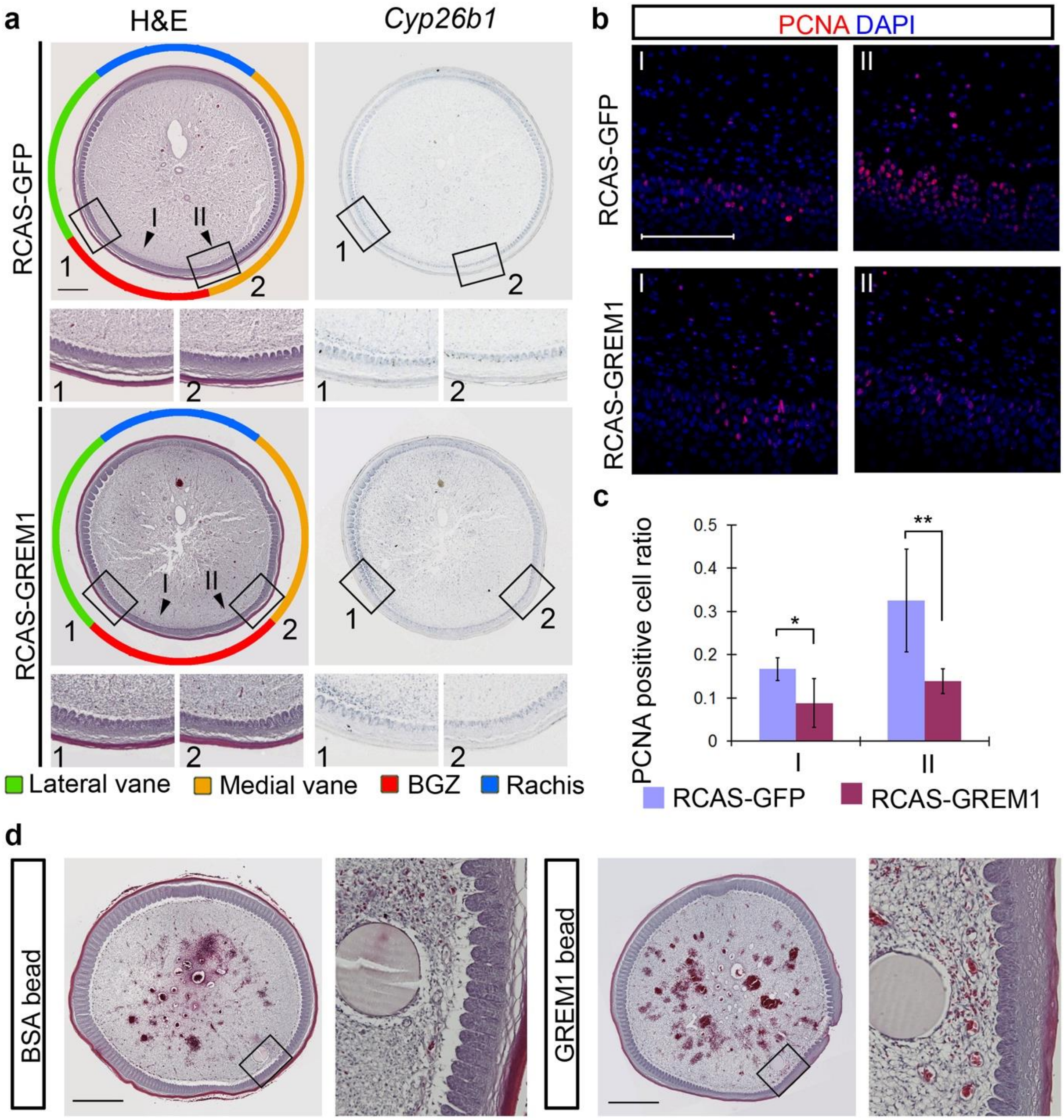

Supplementary Figure 9 | Ectopic *GREM1* induced BGZ fate and decreased cell division.

(**a**) H&E staining and *Cyp26b1 in situ* hybridization of RCAS-GFP, RCAS-GREM1 injected feather follicles, respectively. The boundaries between BGZ and two vanes are highlighted (boxes). Arrowheads indicate Zone 1 and 2 examined in (b). Scale bar: 500 µm. (**b**) Decreased cell proliferation was detected upon *GREM1* mis-expression compared to the control. Scale bar:

100 µm. (**c**) The ratio of PCNA positive cells was calculated by dividing nuclear PCNA positive cell number by the number of DAPI positive cells in regions-of-interest (100 µm diameter circles, $n = 6$ for each zone). *$p < 0.05$, **$p < 0.01$. (**d**) GREM1 soaked beads induced BGZ-like morphology (H&E staining). Scale bar: 500 µm.

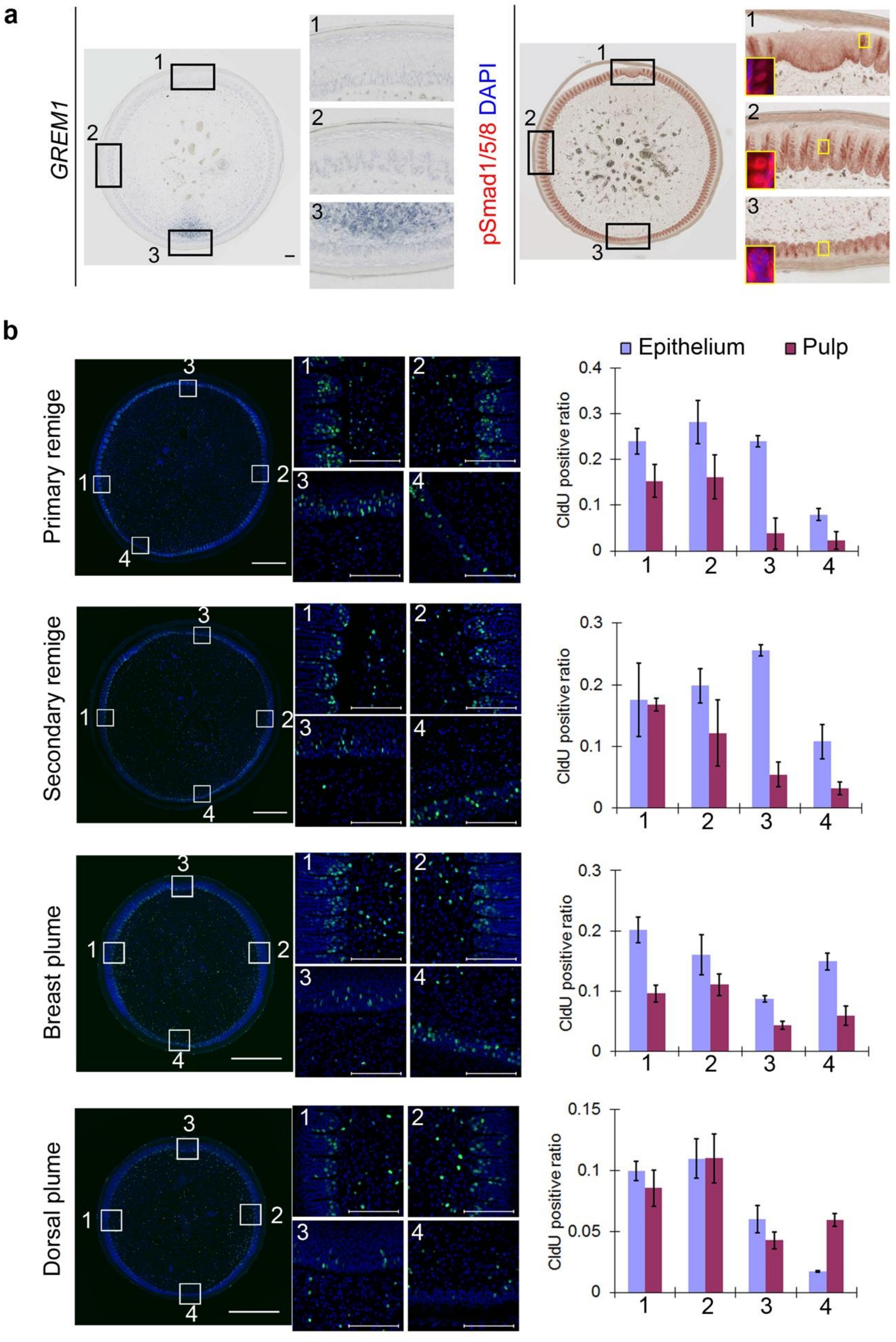
a
GREM1
1
2
3
pSmad1/5/8 DAPI
b
Epithelium
Pulp
Primary remige
Secondary remige
Breast plume
Dorsal plume
CldU positive ratio
0.4
0.3
0.2
0.1
0
0.15
0.05
1
2
3
4

Supplementary Figure 10 | *GREM1* decreases nuclear pSMAD1/5/8 and cell division. (**a**) *GREM1* expression levels are highest in the pulp adjacent to the BGZ where the number of nuclear pSMAD1/5/8 positive cells is the lowest. Black boxes indicate magnified regions. Golden boxes indicate regions magnified to show nuclear pSMAD1/5/8. Scale bar: 100 µm. (**b**) Quantification of proliferating cells (2 h CldU labeling) in the epithelium and pulp corresponding to the two vanes (Zone 1, 2), rachis (Zone 3) and BGZ (Zone 4). 6 Regions of interest (100 µm diameter circles) for statistical analysis in each zone. Scale bar: 500 µm for the leftmost panel, 100µm for the magnified zones. Error bars denote SD.

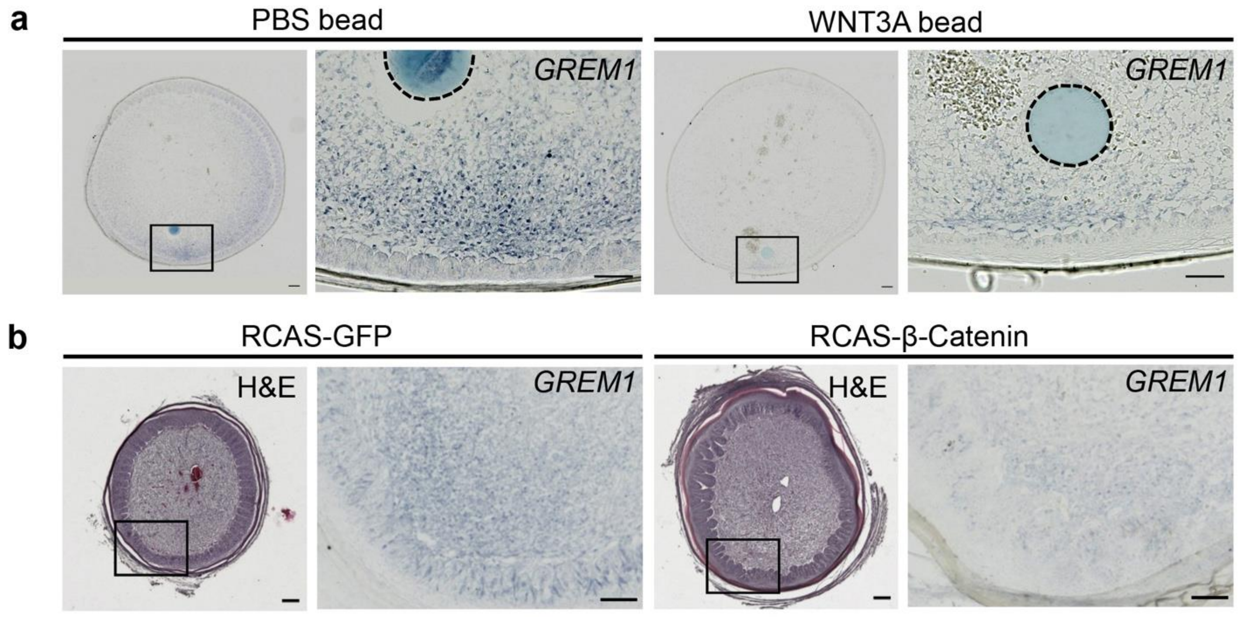

Supplementary Figure 11 | Active WNT signaling inhibits *GREM1* expression. (**a**) Implanting WNT3a (100 µg/ml) soaked beads in the BGZ pulp of growing chicken dorsal plumes reduced *GREM1* expression compared to PBS soaked controls (n = 2, boxes indicate enlarged areas). Scale bar: 100 µm. (**b**) Mis-expressing constitutively active β-catenin downregulated *GREM1* expression compared to the controls (n = 2). Scale bar: 100 µm.

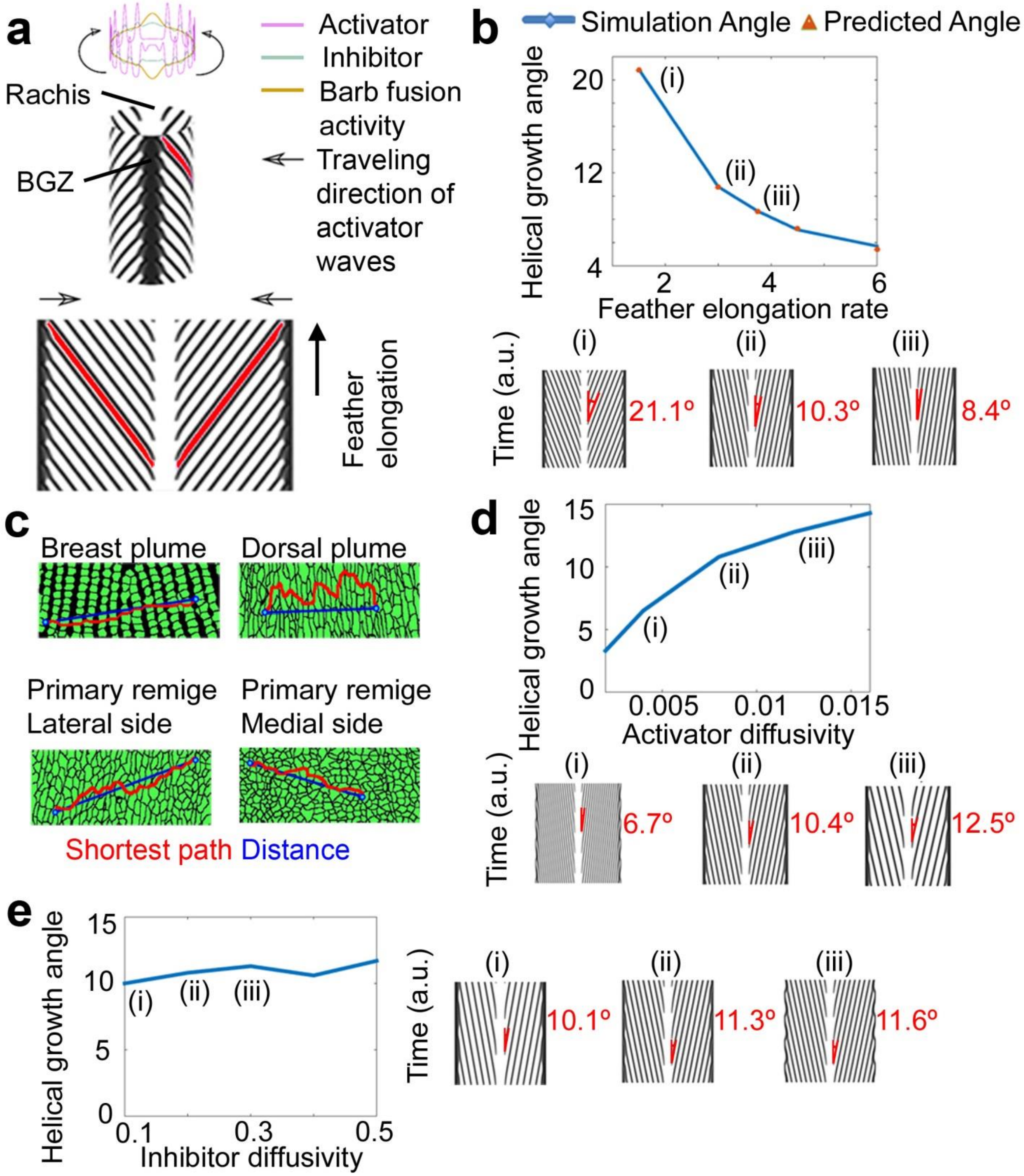

Supplementary Figure 12 | Modeling how epithelial cell shapes affect helical growth angles.

(**a**) Mathematical simulation of a bilateral symmetric feather formation in the cylinder view and flattened view (open up from the BGZ side) based on the previous activator-inhibitor model[18].

The activator waves initialized at the BGZ propagate horizontally to the rachis. Feather elongation occurs along the proximal-distal (vertical) direction. The helical growth angle is an arctangent function of the feather elongation rate divided by the traveling speed of the activator waves. Barbs are highlighted in red. (**b**) Helical growth angle decreases with the increase of gross feather elongation rate. Feather simulations of selected data points are also shown. Simulation angles are simulated using the activator-inhibitor model; predicted angles are calculated by an arctangent function of feather elongation rate over the speed of activator waves (see Methods). (**c**) Differential epithelium tortuosity in different feathers (see Methods). Red lines: shortest path around the cells; blue lines: the distance between two points. Tissue tortuosity is inversely correlated with activator/inhibitor diffusivity. (**d**) Helical growth angle increases with the increase of activator's diffusivity. (**e**) Increasing the inhibitor's diffusivity can only trivially increase the helical growth angle.

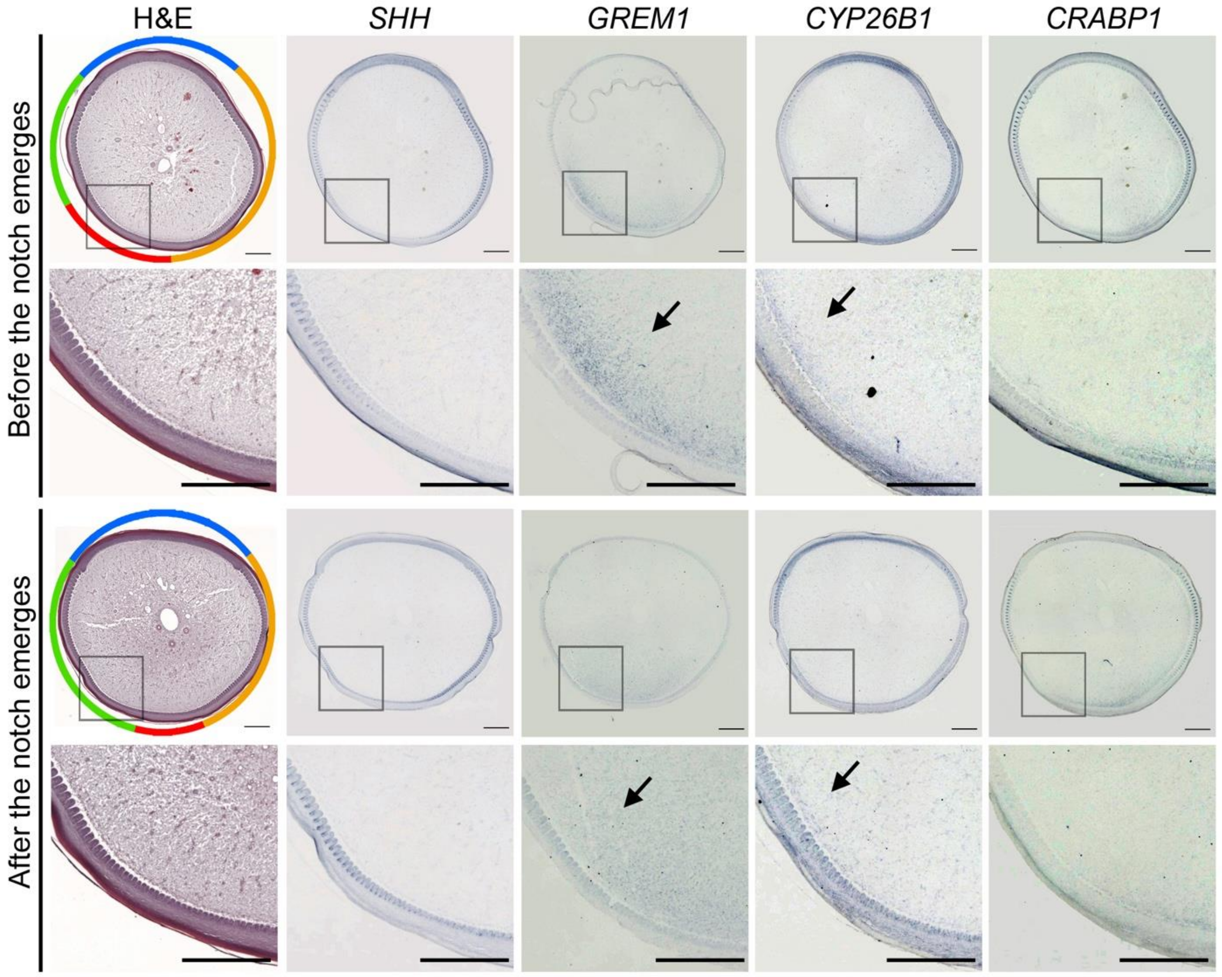

Supplementary Figure 13 | Expression of *GREM1* and *CYP26B1* in emarginated remiges.

H&E staining and *SHH* in situ hybridization demonstrate that part of the BGZ was converted to the lateral vane (the boxed region, which is magnified below) after the vane width increases. During this process *GREM1* and *CYP26b1* expression decrease dramatically while the *CRABP1* expression pattern generally remains unchanged. Arrows indicate enriched *GREM1* and *CYP26b1* expression before the notch emerges which disappears afterward the notch emerges. Scale bars: 500 µm.

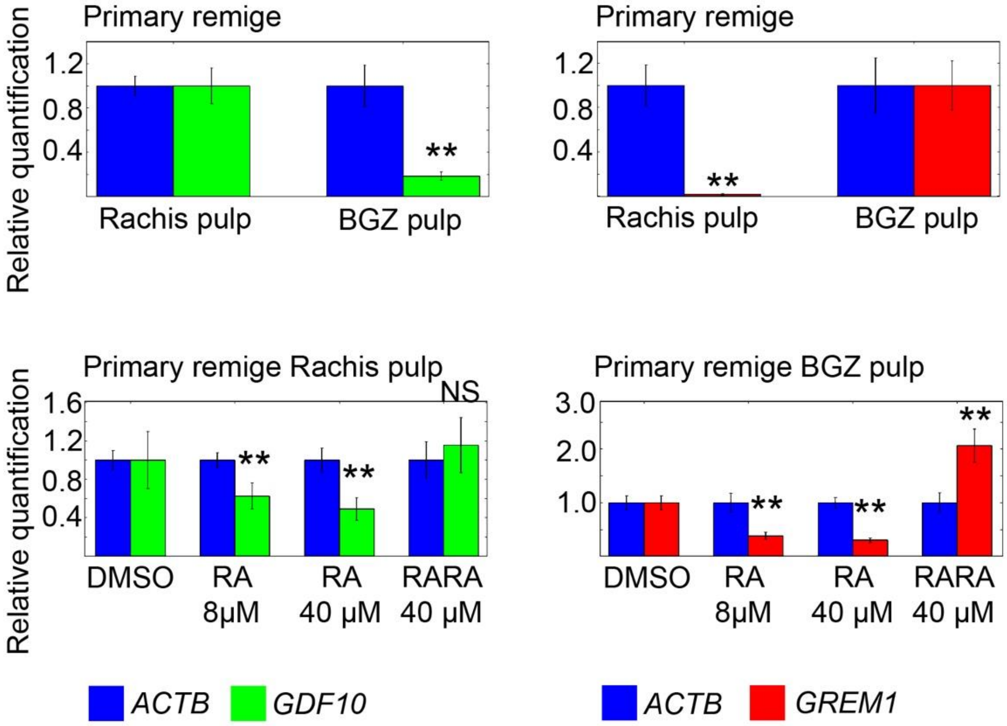

Supplementary Figure 14 | RA signaling downregulates *GDF10* and *GREM1* in pulp cells.

Upper panel: Cultured pulp cells from regions close to the rachis express higher levels of *GDF10* while those from the BGZ pulp express higher levels of *GREM1*. These results fit the *in situ* hybridization data and also indicate the cells can at least temporarily maintain their original gene expression profile *in vitro*. Lower panel: *GDF10* expression in cultured pulp cells from regions adjacent to the rachis and *GREM1* expression in cells from BGZ pulp both decreased in response to RA treatment and increased upon RAR antagonist (RARA: ER50891) treatment. However the increase of *GDF10* upon RAR antagonist treatment is not statistically significant. n = 3 for each condition. Error bars denote SD. ** $p < 0.01$, NS: not significant.

Supplementary Table 1 | Pulp genes negatively correlating with narrow vanes.

Gene Symbol	p-value(Lateral Side vs. Medial Side)	p-value(dorsal plume vs. breast plume)	MeanRatio(Lateral Side vs. Medial Side)	MeanRatio (dorsal plume vs. breast plume)	FoldChange (Lateral Side vs. Medial Side)	FoldChange (dorsal plume vs. breast plume)	FoldChange (Lateral Side vs. Medial Side) (Description)	FoldChange (dorsal plume vs. breast plume) (Description)
CRABP-I	0.013257	0.001565	0.517303	0.097848	-1.9331	-10.22	Lateral Side down vs Medial Side	dorsal down vs breast
CSTA	0.179635	0.20982	0.173737	0.261235	-5.75583	-3.82796	Lateral Side down vs Medial Side	dorsal down vs breast
DSP	0.118802	0.246211	0.470314	0.449726	-2.12624	-2.22358	Lateral Side down vs Medial Side	dorsal down vs breast
ENSGALG00000004747	0.026436	0.245977	0.405999	0.389917	-2.46306	-2.56465	Lateral Side down vs Medial Side	dorsal down vs breast
ENSGALG00000017398	0.231916	0.21309	0.547702	0.486084	-1.82581	-2.05726	Lateral Side down vs Medial Side	dorsal down vs breast
ENSGALG00000026754	0.362004	0.324221	0.522169	0.151299	-1.91509	-6.60942	Lateral Side down vs Medial Side	dorsal down vs breast
ENSGALG00000028374	0.356533	0.23006	0.258447	0.276811	-3.86927	-3.61257	Lateral Side down vs Medial Side	dorsal down vs breast
ENSGALG00000029144	0.123266	0.24257	0.193918	0.154143	-5.15681	-6.48749	Lateral Side down vs Medial Side	dorsal down vs breast
FABP5	0.242121	0.220834	0.361231	0.295303	-2.76831	-3.38636	Lateral Side down vs Medial Side	dorsal down vs breast
FGF10	0.060994	0.0042	0.425766	0.113957	-2.34871	-8.77526	Lateral Side down vs Medial Side	dorsal down vs breast
GDF10	0.095151	0.019027	0.464264	0.026961	-2.15395	-37.0908	Lateral Side down vs Medial Side	dorsal down vs breast
KRT8	0.087444	0.105137	0.526848	0.504467	-1.89808	-1.98229	Lateral Side down vs Medial Side	dorsal down vs breast
LAMC3	0.392246	0.011696	0.534043	0.118991	-1.87251	-8.40402	Lateral Side down vs Medial Side	dorsal down vs breast
MSX2	0.076114	0.246859	0.515468	0.395452	-1.93998	-2.52875	Lateral Side down vs Medial Side	dorsal down vs breast
PKP1	0.13455	0.27828	0.48228	0.427192	-2.07348	-2.34087	Lateral Side down vs Medial Side	dorsal down vs breast
PROCR	0.288164	0.006739	0.502317	0.315073	-1.99077	-3.17387	Lateral Side down vs Medial Side	dorsal down vs breast
STK32A	0.069882	0.058007	0.458529	0.543458	-2.18089	-1.84007	Lateral Side down vs Medial Side	dorsal down vs breast
VTN	0.013344	0.002659	0.549993	0.141826	-1.8182	-7.05087	Lateral Side down vs Medial Side	dorsal down vs breast

Supplementary Table 2 | Pulp genes positively correlating with narrow vanes.

Gene Symbol	p-value(Lateral Side vs. Medial Side)	p-value(dorsal plume vs. breast plume)	MeanRatio(Lateral Side vs. Medial Side)	MeanRatio (dorsal plume vs. breast plume)	FoldChange (Lateral Side vs. Medial Side)	FoldChange (dorsal plume vs. breast plume)	FoldChange (Lateral Side vs. Medial Side) (Description)	FoldChange (dorsal plume vs. breast plume) (Description)
ASIP	0.124132	0.06	2.24099	3.01047	2.24099	3.01047	Lateral Side up vs Medial Side	dorsal up vs breast
CYP26B1	0.228511	0.00209	2.97553	2.63736	2.97553	2.63736	Lateral Side up vs Medial Side	dorsal up vs breast
ENSGALG00000016251	0.001543	0.001368	2.5338	4.06433	2.5338	4.06433	Lateral Side up vs Medial Side	dorsal up vs breast
ENSGALG00000019852	0.017912	0.001801	4.14026	129.776	4.14026	129.776	Lateral Side up vs Medial Side	dorsal up vs breast
ENSGALG00000027770	0.019064	0.032245	5.7348	732154	5.7348	732154	Lateral Side up vs Medial Side	dorsal up vs breast
EYA2	0.003905	0.002502	1.90395	9.19384	1.90395	9.19384	Lateral Side up vs Medial Side	dorsal up vs breast
FBLN5	0.070538	0.034725	2.07429	3.64168	2.07429	3.64168	Lateral Side up vs Medial Side	dorsal up vs breast
FST	0.056628	0.000803	1.92726	4.67716	1.92726	4.67716	Lateral Side up vs Medial Side	dorsal up vs breast
GREM1	0.03835	0.04425	7.29995	2.12391	7.29995	2.12391	Lateral Side up vs Medial Side	dorsal up vs breast
LRIG1	0.067694	0.009156	2.30657	2.5807	2.30657	2.5807	Lateral Side up vs Medial Side	dorsal up vs breast
P37NB	0.13759	0.005831	2.12539	4.46892	2.12539	4.46892	Lateral Side up vs Medial Side	dorsal up vs breast
PKIA	0.0126	0.012188	1.86006	2.90837	1.86006	2.90837	Lateral Side up vs Medial Side	dorsal up vs breast
SCG5	0.047521	0.023776	4.78823	4.54587	4.78823	4.54587	Lateral Side up vs Medial Side	dorsal up vs breast
SLC9A7	0.204746	0.001142	2.00464	2.9594	2.00464	2.9594	Lateral Side up vs Medial Side	dorsal up vs breast
SMIM5	0.035731	0.045536	2.01817	2.13812	2.01817	2.13812	Lateral Side up vs Medial Side	dorsal up vs breast

Supplementary Table 3 | Epithelial genes negatively correlating with narrow vanes.

Gene Symbol	p-value(Lateral Side vs. Medial Side)	p-value(dorsal plume vs. breast plume)	MeanRatio(Lateral Side vs. Medial Side)	MeanRatio (dorsal plume vs. breast plume)	FoldChange (Lateral Side vs. Medial Side)	FoldChange (dorsal plume vs. breast plume)	FoldChange (Lateral Side vs. Medial Side) (Description)	FoldChange (dorsal plume vs. breast plume) (Description)
AP1S3	0.071522	0.00557	0.530709	0.124578	-1.88427	-8.02712	Lateral Side down vs Medial Side	dorsal down vs breast
CA13	0.293588	0.00059	0.555201	0.134941	-1.80115	-7.41066	Lateral Side down vs Medial Side	dorsal down vs breast
ENSGALG00000006133	0.005429	0.000174	0.273434	0.006083	-3.65719	-164.384	Lateral Side down vs Medial Side	dorsal down vs breast
ENSGALG00000024141	0.092004	0.000266	0.372151	0.017268	-2.68708	-57.9105	Lateral Side down vs Medial Side	dorsal down vs breast
ENSGALG00000025726	0.214317	7.32E-06	0.403345	0.00391	-2.47927	-255.732	Lateral Side down vs Medial Side	dorsal down vs breast
ENSGALG00000025863	0.288782	0.000862	0.326542	0.057756	-3.0624	-17.3143	Lateral Side down vs Medial Side	dorsal down vs breast
ENSGALG00000025900	0.028151	0.000798	0.506569	0.022023	-1.97406	-45.4069	Lateral Side down vs Medial Side	dorsal down vs breast
ENSGALG00000025985	0.516686	0.000168	0.428527	0.017591	-2.33358	-56.8474	Lateral Side down vs Medial Side	dorsal down vs breast
ENSGALG00000026219	0.108547	0.001381	0.33462	0.009213	-2.98846	-108.547	Lateral Side down vs Medial Side	dorsal down vs breast
ENSGALG00000026592	0.362851	0.440068	0.278414	0.532468	-3.59177	-1.87805	Lateral Side down vs Medial Side	dorsal down vs breast
ENSGALG00000026660	0.394246	0.022736	0.282541	0.080784	-3.53931	-12.3788	Lateral Side down vs Medial Side	dorsal down vs breast
ENSGALG00000026987	0.215335	0.000191	0.434367	0.036508	-2.3022	-27.391	Lateral Side down vs Medial Side	dorsal down vs breast
ENSGALG00000027167	0.118545	0.000804	0.549109	0.016775	-1.82113	-59.6124	Lateral Side down vs Medial Side	dorsal down vs breast
ENSGALG00000027679	0.012988	0.003446	0.505696	0.112649	-1.97747	-8.87711	Lateral Side down vs Medial Side	dorsal down vs breast
ENSGALG00000027859	0.025731	0.000303	0.304379	0.00504	-3.28538	-198.409	Lateral Side down vs Medial Side	dorsal down vs breast
ENSGALG00000028192	0.128874	0.000203	0.36141	0.021709	-2.76694	-46.0635	Lateral Side down vs Medial Side	dorsal down vs breast
ENSGALG00000028211	0.170467	0.000238	0.376689	0.037407	-2.65471	-26.7328	Lateral Side down vs Medial Side	dorsal down vs breast
ENSGALG00000028287	0.141015	0.004354	0.331529	0.148906	-3.01633	-6.71564	Lateral Side down vs Medial Side	dorsal down vs breast

ENSGALG00000028366	0.341402	0.002662	0.396264	0.152326	-2.52357	-6.56487	Lateral Side down vs Medial Side	dorsal down vs breast
ENSGALG00000028371	0.338846	0.480844	0.389958	0.552322	-2.56438	-1.81054	Lateral Side down vs Medial Side	dorsal down vs breast
ENSGALG00000028843	0.036415	0.001686	0.448153	0.040031	-2.23138	-24.9808	Lateral Side down vs Medial Side	dorsal down vs breast
F-KER	0.043291	0.000479	0.394476	0.031371	-2.53501	-31.8763	Lateral Side down vs Medial Side	dorsal down vs breast
FK21	0.195268	0.002084	0.338759	0.033087	-2.95195	-30.2237	Lateral Side down vs Medial Side	dorsal down vs breast
FK27	0.367287	0.004976	0.315145	0.090281	-3.17314	-11.0766	Lateral Side down vs Medial Side	dorsal down vs breast
JAC	0.261847	0.030227	0.48121	0.39716	-2.07809	-2.51788	Lateral Side down vs Medial Side	dorsal down vs breast
PADI1	0.045967	0.001381	0.457927	0.060391	-2.18375	-16.5589	Lateral Side down vs Medial Side	dorsal down vs breast
SCEL	0.062087	0.012171	0.542305	0.351796	-1.84398	-2.84256	Lateral Side down vs Medial Side	dorsal down vs breast
TNFRSF19	0.057703	0.002839	0.515489	0.375954	-1.93991	-2.6599	Lateral Side down vs Medial Side	dorsal down vs breast
TXNRD1	0.084353	0.003413	0.458893	0.1505	-2.17916	-6.64452	Lateral Side down vs Medial Side	dorsal down vs breast

Supplementary Table 4 | Epithelial genes positively correlating with narrow vanes.

Gene Symbol	p-value(Lateral Side vs. Medial Side)	p-value(dorsal plume vs. breast plume)	MeanRatio(Lateral Side vs. Medial Side)	MeanRatio(dorsal plume vs. breast plume)	FoldChange(Lateral Side vs. Medial Side)	FoldChange(dorsal plume vs. breast plume)	FoldChange(Lateral Side vs. Medial Side) (Description)	FoldChange(dorsal plume vs. breast plume) (Description)
APOA1	0.241196	0.010203	2.38605	5.28519	2.38605	5.28519	Lateral Side up vs Medial Side	dorsal up vs breast

Supplementary Table 5 | RARE within 13 kb region upstream of chicken *GREM1* locus.

DR1-1 in Fig. 6c is in the first row (highest conservativeness in chicken, turkey and zebra finch).

Detailed Matrix Information	Opt.	Start position	End position	Anchor position	Strand	Core sim.	Matrix sim.	Mat. sim. - opt.	conservativeness
Retinoic acid receptor / retinoid X receptor heterodimer, DR1 sites	0.78	261	285	273	+	1	0.811	0.031	96%
Retinoic acid receptor / retinoid X receptor heterodimer, DR1 sites	0.78	3269	3293	3281	+	1	0.821	0.041	no
Retinoic acid receptor / retinoid X receptor heterodimer, DR1 sites	0.78	3656	3680	3668	+	1	0.848	0.068	no
Retinoic acid receptor gamma, homodimer DR2 binding site	0.84	4286	4310	4298	-	1	0.912	0.072	no
Retinoic acid receptor / retinoid X receptor heterodimer, DR5 sites	0.81	4337	4361	4349	+	0.86	0.854	0.044	no
Retinoic acid receptor / retinoid X receptor heterodimer, DR1 sites	0.78	7679	7703	7691	-	1	0.812	0.032	no
Retinoic acid receptor gamma, homodimer DR2 binding site	0.84	8991	9015	9003	+	1	0.857	0.017	no
Retinoic acid receptor gamma, homodimer DR2 binding site	0.84	9690	9714	9702	-	1	0.866	0.026	no
Retinoic acid receptor / retinoid X receptor heterodimer, DR5 sites	0.81	9970	9994	9982	-	0.86	0.888	0.078	no
Retinoic acid receptor gamma, homodimer DR2 binding site	0.84	11417	11441	11429	-	1	0.915	0.075	no
Retinoic acid receptor gamma, homodimer DR2 binding site	0.84	12409	12433	12421	+	1	0.847	0.007	no
Retinoic acid receptor gamma, homodimer DR2 binding site	0.84	12540	12564	12552	+	1	0.852	0.012	no

Supplementary Table 6 | RARE within 13 kb region downstream of chicken *GREM1* locus.

DR1-2 in Fig. 6c is in the third row (highest conservativeness in chicken, turkey and zebra finch)**.**

Detailed Matrix Information	Opt.	Start position	End position	Anchor position	Strand	Core sim.	Matrix sim.	Mat. sim. - opt.	Conservativeness
Retinoid X receptor homodimer, DR1 sites	0.78	767	791	779	+	0.889	0.784	0.004	92%
Retinoic acid receptor gamma, homodimer DR2 binding site	0.84	2752	2776	2764	-	1	0.852	0.012	no
Retinoic acid receptor / retinoid X receptor heterodimer, DR1 sites	0.78	3121	3145	3133	-	1	0.814	0.034	95%
Retinoic acid receptor / retinoid X receptor heterodimer, DR1 sites	0.78	3277	3301	3289	+	1	0.808	0.028	89%
Retinoic acid receptor gamma, homodimer DR2 binding site	0.84	4213	4237	4225	+	1	0.935	0.095	no
Retinoic acid receptor / retinoid X receptor heterodimer, DR1 sites	0.78	5010	5034	5022	+	1	0.809	0.029	no
Retinoic acid receptor gamma, homodimer DR2 binding site	0.84	5560	5584	5572	+	1	0.917	0.077	no
Retinoic acid receptor / retinoid X receptor heterodimer, DR5 sites	0.81	6698	6722	6710	+	1	0.818	0.008	no
Retinoic acid receptor / retinoid X receptor heterodimer, DR1 sites	0.78	7325	7349	7337	+	1	0.795	0.015	no
Retinoic acid receptor / retinoid X receptor heterodimer, DR1 sites	0.78	7377	7401	7389	-	1	0.793	0.013	no
Retinoic acid receptor gamma, homodimer DR2 binding site	0.84	7386	7410	7398	+	1	0.874	0.034	no
Retinoic acid receptor / retinoid X receptor heterodimer, DR5 sites	0.81	10650	10674	10662	-	1	0.827	0.017	no
Retinoic acid receptor gamma, homodimer DR2 binding site	0.84	11360	11384	11372	+	1	0.865	0.025	no

Supplementary Table 7. Parameters for the model of Periodic branching pattern.

**Parameter**	**Value**
D_A	[8e-3, 2e-2]
D_B	[0.2, 0.5]
r_A	0.01
r_B	0.015
r_C	3e-4
b_A	[0, 1e-3]
b_B	[0.15, 0.3]
b_C	5e-4
s	1e-2
s_A	1.8
s_B	1.8
s_C	0.35
s_G	1e-3
n_G	-2

Supplementary Table 8. Parameters for the estimation of tissue tortuosity.

Feather	Mean Tortuosity (a.u.)	D : D* (a.u.)
Breast contour	1.13	1 : 0.78
Dorsal contour	1.61	1 : 0.39
Primary remige (lateral)	1.34	1 : 0.56
Primary remige (medial)	1.18	1 : 0.72

Supplementary Table 9. Parameters for MRF model.

**Parameter**	**Value**
D_{RAo}	[1e-2, 1e-1]
D_{WNT}	1e-2
D_{GDF}	1e-2
D_{GREM}	1e-2
V_{RAo}	[0, 1e-5]
V_{R}	2e-6
V_{GREM}	1e-6
B_{RAo}	1e-1
B_{WNT}	0.1
B_{GDF}	0
B_{GREM}	0
B_{CYP}	[0, 1e-1]
B_{BP}	[0, 1e-1]
r_{RAi1}	1
r_{R1}	1e-4
r_{R2}	1e-5
r_{BP1}	1e-4
r_{BP2}	1e-5
r_{RAi2}	1e-1
r_{WNT}	1e-4
r_{GDF}	1e-4
r_{GREM}	1e-4
k_{on}	1e-3
k_{off}	1e-6
m_{on}	1
m_{off}	1e-3
j_{α}	1
j_{β}	1e-3
k_{p}	5e-1
k_{1}	1e-2
k_{2}	1e-1
k_{3}	4e-3
k_{4}	5e-5
k_{5}	1e-1
n_{1}	2
n_{2}	1
n_{3}	-1
n_{4}, n_{5}	-2
β	[0, .5]
v_{cyp}	[1, 20]
v_{bp}	[1, 5]
v_{wnt}	[1, 5]

v_{gdf}	[1, 20]
a_{cyp}	[0, 15]
a_{bp}	[0, 1]
a_{wnt}	1
a_{gdf}	1
